# MXenes Contacts for *p*‐type 2D Electronics

**DOI:** 10.1002/advs.76591

**Published:** 2026-08-03

**Authors:** Tianchao Guo, Maolin Chen, Yizhou Wang, Chen Liu, Xiangming Xu, Linqu Luo, Dekang Zhu, Ning Chu, Xiaowen Zhang, Alfonso Caraveo, Thomas D. Anthopoulos, Xixiang Zhang, Husam N. Alshareef

**Affiliations:** ^1^ Materials Science and Engineering Physical Science and Engineering Division King Abdullah University of Science and Technology (KAUST) Thuwal Saudi Arabia; ^2^ Center for Renewable Energy and Storage Technology (CREST) King Abdullah University of Science and Technology (KAUST) Thuwal Saudi Arabia; ^3^ Shanghai Institute of Microsystem and Information Technology Chinese Academy of Sciences Shanghai China; ^4^ Photon Science Institute Henry Royce Institute Department of Electrical and Electronic Engineering the University of Manchester Manchester UK

**Keywords:** 2D materials, MXenes, p‐type transistors, solution‐processed

## Abstract

MXenes, a family of two‐dimensional (2D) transition‐metal carbides and nitrides, offer a compelling combination of metallic conductivity and tunable surface chemistry. However, their potential as electrical contacts for *p*‐type 2D semiconductors remains largely untapped. Here, we systematically investigate the role of MXene composition by comparing Ti_3_C_2_T_x_, Nb_2_CT_x_, and Mo_2_CT_x_ as contacts for *p*‐type 2H‐MoTe_2_ transistors. Through a polymer‐assisted transfer process that ensures high‐quality interfaces, we identify Nb_2_CT_x_ as the best‐performing contact material among the MXenes examined in this work, attributed to its superior band alignment, which markedly reduces contact resistance compared to both conventional metals and other MXenes. Consequently, Nb_2_CT_x_/2H‐MoTe_2_ field‐effect transistors achieve a high hole mobility of approximately 17 cm^2^V^−1^s^−1^ and an on/off current ratio exceeding 10^3^. Temperature‐dependent measurements further reveal a near‐ideal interface, with a Schottky barrier height of only a few millielectronvolts under strong gate bias. These findings establish compositional engineering of MXenes as a powerful strategy for designing electrode–semiconductor interfaces and position Nb_2_CT_x_ as a scalable, high‐performance contact material for advancing *p*‐type 2D electronics.

## Graphical abstract

Solution‐processed MXene contacts are engineered for *p*‐type 2H‐MoTe_2_ transistors. By comparing Ti_3_C_2_T_x_, Nb_2_CT_x_, and Mo_2_CT_x_, Nb_2_CT_x_ is identified as the best‐performing contact among the MXenes examined in this work, offering favorable work‐function alignment, low contact resistance, and near‐ohmic hole injection for scalable 2D electronics.

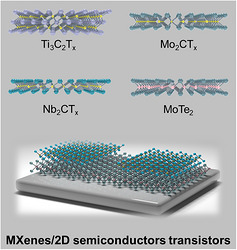

## 1 Introduction

Since their discovery in 2011, MXenes have rapidly emerged as a major research frontier in materials science and nanotechnology [1, 2, 3, 4, 5]. These two‑dimensional (2D) metal carbides and nitrides combine exceptional electrical conductivity, mechanical flexibility, and tunable surface chemistry, giving them broad potential in applications ranging from energy storage and sensing to next‑generation electronic devices [6, 7, 8]. For electronic applications in particular, Ti_3_C_2_T_x_ stands out due to its highly mature fabrication protocols. It has already been used as electrode materials across a wide spectrum of semiconductors, including oxide semiconductors [9], organic semiconductors [10, 11], quantum dots [12], and transition‐metal dichalcogenides (TMDs) [13, 14]. This track record demonstrates the strong potential of MXenes as scalable, high‐performance contact materials.

To leverage these advantages, it is compelling to consider the role of MXenes as contact materials for emerging semiconductor platforms. Among them, 2D TMDs have garnered considerable attention owing to their distinctive properties and broad prospects in electronics [15, 16, 17, 18]. The performance of TMD‑based devices critically depends on the quality of the electrode–semiconductor interface [19, 20]. For *n*‑type TMDs, low‑work‑function (*W_F_*) metals such as In and Bi afford favorable band alignment and permit low‐energy deposition that preserves the interface [19]. By contrast, *p*‑type TMDs require high‑*W_F_* metals such as Pt, Pd, and Au to achieve optimal band alignment. However, their high melting points (>1000°C) necessitate high‐energy deposition methods. These often cause significant interface damage, which offsets the benefits of *W_F_* alignment and degrades device performance [20].

**FIGURE 1:**
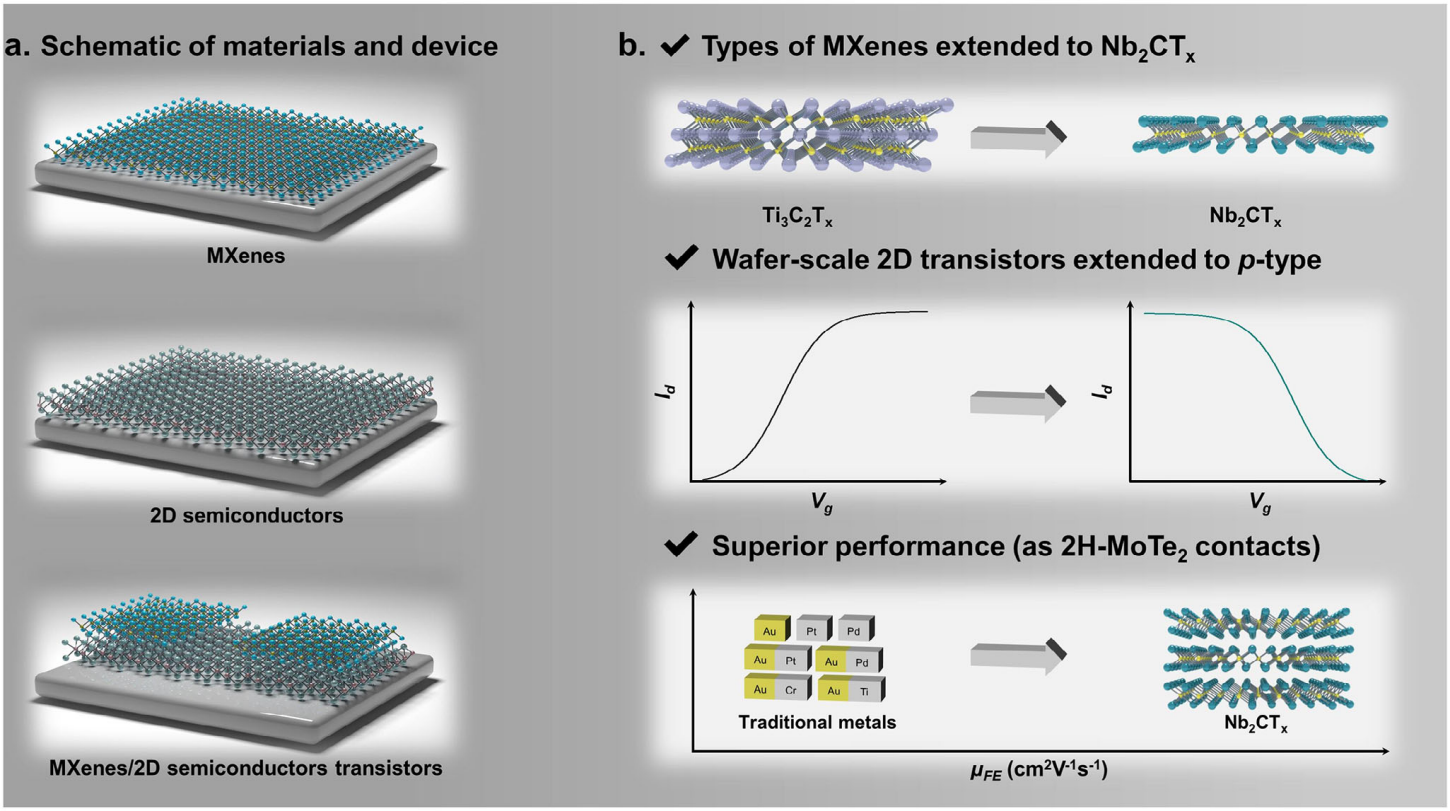
Schematics of MXene/*p*‐2D semiconductor transistors. (a) Schematic illustration of the MXenes, 2D semiconductors, and MXenes/2D semiconductors transistors. (b) Extension of MXenes to Nb_2_CT_x_ enabling *p*‐type 2D transistors with superior contact performance over traditional metals [26, 27, 28, 29, 30, 31, 32, 33].

Given the limitations of conventional high‑*W_F_* metals in forming low‑damage contacts with *p*‑type TMDs, MXenes have recently emerged as a promising new class of electrode materials for *p*‑type 2D semiconductor transistors. Owing to their excellent solution processability, MXenes can be deposited under mild, low‑cost conditions, thereby minimizing interface damage compared with traditional vacuum‑deposited high‑*W_F_* metals. In addition, the *W_F_* of MXenes can be widely tuned via surface termination engineering and compositional control, enabling optimized band alignment and reduced contact resistance with *p*‑type 2D semiconductors [21, 22, 23]. These combined advantages—low‑energy deposition, low cost, *W_F_* tunability, and structural versatility—make MXenes highly attractive for improving the performance of 2D‑material‑based electronics. Nevertheless, systematic, device‐level studies of MXenes/*p*‐type 2D semiconductor contacts, particularly in practical transistor configurations (Figure 1a), remain scarce.

Here, we systematically investigate MXene films as electrodes for high‑performance *p*‑type 2D transistors, using 2H‐MoTe_2_ as a representative *p*‐type 2D semiconductor. Building upon prior work that predominantly employed Ti_3_C_2_T_x_, we selected Ti_3_C_2_T_x_ as a benchmark MXene because it is the most extensively studied member of the MXene family and has been widely explored as an electrode/contact material in transistor‐related studies. To extend the compositional space beyond Ti‐based MXenes, Nb_2_CT_x_, and Mo_2_CT_x_ were further chosen because they are synthetically mature and solution‐processable. Previous studies have suggested that the work function ranges of Nb_2_CT_x_ and Mo_2_CT_x_ differ from Ti_3_C_2_T_x_ [14, 23, 24, 25], enriching the compositional space for contact engineering (Figure 1b). In parallel, we broaden the scope of MXene‑based 2D transistors from the conventional *n*‑type domain to *p*‑type channels. Leveraging solution‑processed MXene films and a reliable transfer process, we achieve high‑quality MXene/2H‐MoTe_2_ interfaces. Electrical measurements reveal that 2H‐MoTe_2_ transistors with Nb_2_CT_x_ contacts deliver markedly superior performance—achieving an average field‐effect hole mobility (*µ_FE_*) of ∼17  cm^2^ V^−1^ s^−1^ and an on/off (*I_on_/I_off_*) ratio exceeding 10^3—^surpassing not only devices with Ti_3_C_2_T_x_ and Mo_2_CT_x_ contacts, but also those employing traditional high‑*W_F_* metal electrodes (Table S1). These results underscore the critical role of MXene composition in optimizing *p*‑type 2D contacts and point to a cost‑effective, scalable pathway toward high‑performance 2H‐MoTe_2_‑based and other 2D electronic devices.

## 2 Characterization of MXenes and 2H‐MoTe_2_

**FIGURE 2:**
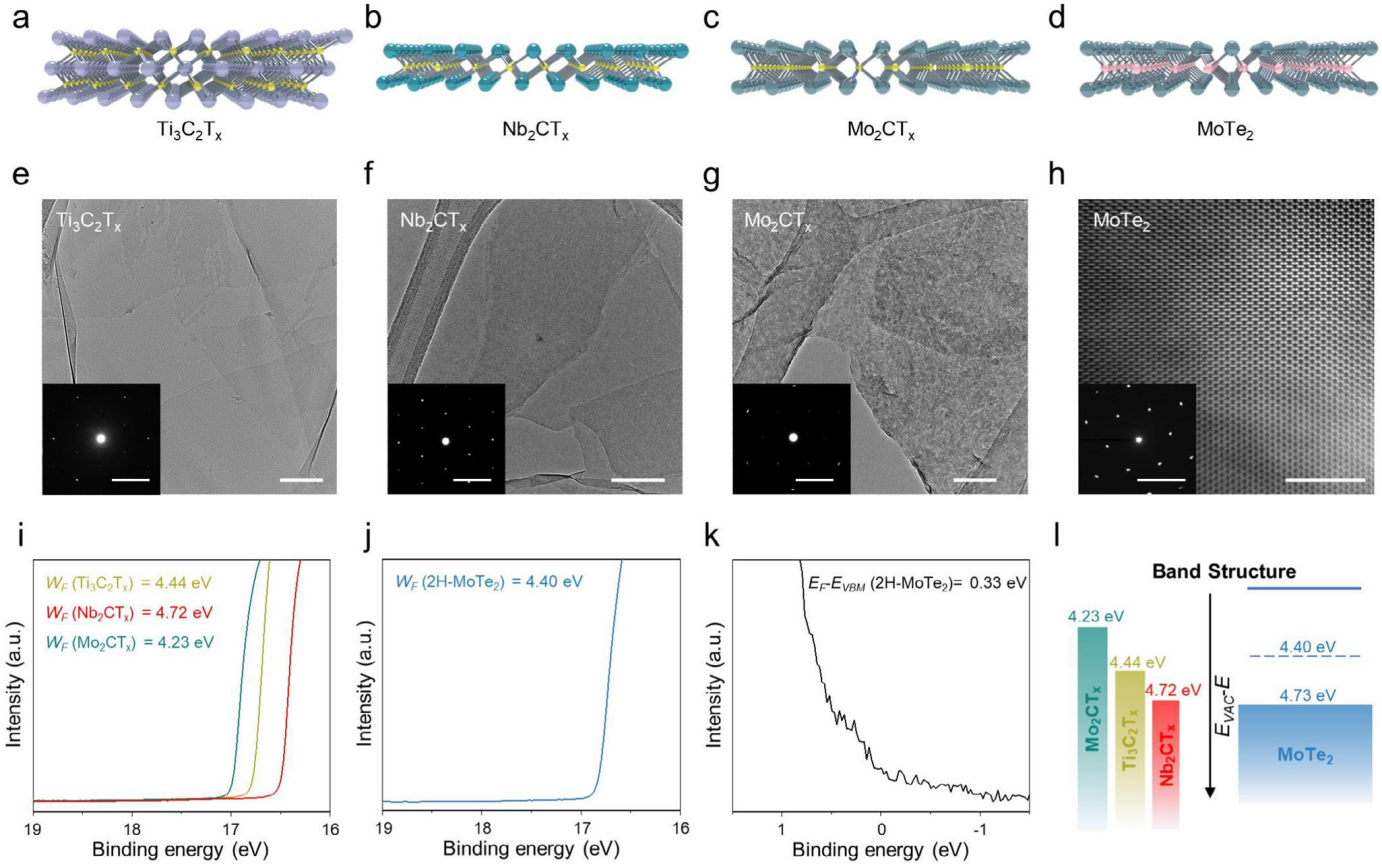
Structural, morphological, and electronic characterizations of MXenes and 2H‐MoTe_2_. (a–d) Crystal structure models of (a) Ti_3_C_2_T_x_, (b) Nb_2_CT_x_, (c) Mo_2_CT_x_, and (d) 2H‐MoTe_2_. (e–h) TEM images of MXene nanosheets and a high‑resolution STEM image of 2H‐MoTe_2_, with corresponding SAED patterns (insets): (e) Ti_3_C_2_T_x_, (f) Nb_2_CT_x_, (g) Mo_2_CT_x_, and (h) 2H‐MoTe_2_. Scale bars are 100 nm (e–g, main), 5 nm (h, main), and 5 1/nm (all insets). (i) UPS secondary electron cutoff spectra of the three MXene films. (j) UPS secondary electron cutoff spectrum of 2H‐MoTe_2_. (k) UPS valence‑band spectrum of 2H‐MoTe_2_. (l) Schematic band alignment diagram of MXenes and 2H‐MoTe_2_ based on UPS data.

Figure 2 summarizes the structural, morphological, and electronic properties of three representative MXenes—Ti_3_C_2_T_x_, Nb_2_CT_x_, and Mo_2_CT_x—_and the *p*‑type semiconductor 2H‐MoTe_2_, which serve as the contact electrodes and channel material, respectively. The crystal structures of the four materials are illustrated in Figure 2a–d. The three MXenes were synthesized by selective etching of their corresponding MAX precursors (Ti_3_AlC_2_, Nb_2_AlC, and Mo_2_Ga_2_C) (Figure S1), followed by chemical intercalation and exfoliation into few‑layer nanosheets (Figure S2, see **Methods**). For Nb_2_AlC and Mo_2_Ga_2_C, hydrofluoric acid (HF) was used as the etchant, while a mixture of HF and hydrochloric acid (HCl) was employed for Ti_3_AlC_2_. Subsequent intercalation effectively separated the layers, yielding ultrathin flakes dispersed in aqueous solutions. The 2H‐MoTe_2_ thin films were synthesized by a two‑step process involving magnetron sputtering of Mo onto SiO_2_/Si substrates, followed by CVD tellurization to obtain the semiconducting 2H phase (Figure S3) [34].

Figure 2e–h present representative electron microscopy images and corresponding selected‑area electron diffraction (SAED) patterns. The transmission electron microscopy (TEM) images of Ti_3_C_2_T_x_, Nb_2_CT_x_, and Mo_2_CT_x_ confirm their characteristic two‑dimensional flake morphology, while the SAED patterns reveal hexagonal diffraction spots, indicating high crystallinity and ordered in‑plane atomic arrangements. Complementary atomic force microscopy (AFM) measurements further verify the ultrathin, layered nature of these MXene flakes (Figure S4). For 2H‐MoTe_2_, the atomic‑resolution scanning transmission electron microscopy (STEM) image (Figure 2h) clearly displays a well‑defined layered structure, and its SAED pattern further verifies the hexagonal crystal lattice.

The phase purity, structural integrity, and surface chemistry of the four materials were characterized using Raman spectroscopy, x‐ray photoelectron spectroscopy (XPS), and x‐ray diffraction (XRD) (Figures S5–S7). Raman spectra (Figure S5) confirm the successful synthesis of the target materials. Ti_3_C_2_T_x_ and Nb_2_CT_x_ show prominent A_1g_ peaks, while Mo_2_CT_x_ displays both A_1g_ and E_2g_ modes, consistent with reported MXene signatures [35, 36, 37, 38]. Notably, no apparent oxidation‑related peaks were detected in any of the MXene samples, indicating that the materials remained chemically stable during processing and characterization. The 2H‐MoTe_2_ sample exhibits distinct E_2g_ and A_1g_ modes, with no signs of 1T′ or secondary phases, thereby verifying its semiconducting nature [39]. High‐resolution XPS (Figure S6) provides further validation of the surface chemistry. For Ti_3_C_2_T_x_, the Ti 2p spectrum shows components from Ti–C, Ti^2+^, and Ti^3+^, along with a minor TiO_2_ contribution. Nb_2_CT_x_ exhibits dominant Nb^5+^ peaks, while Mo_2_CT_x_ displays a mixture of Mo^4+^ and Mo^5+^ species, in agreement with previously reported MXene surface chemistries [38, 40, 41, 42]. In the case of 2H‐MoTe_2_ (Figure S6d), the Mo 3d peaks are mainly assigned to Mo^4^^+^, consistent with the expected valence state [39]. A weak Mo^5^^+^ shoulder likely arises from slight surface oxidation during sample storage or air exposure, as commonly observed in telluride materials [33, 43]. Finally, XRD analysis (Figure S7) of the MXene films confirms the absence of impurities and secondary phases, indicating that the MAX precursor phase has been successfully removed. The 2H‐MoTe_2_ film presents sharp diffraction peaks that are indexed to the 2H phase, confirming its high crystallinity and phase purity [26]. Collectively, these data confirm that all four materials were successfully synthesized with the intended structural and chemical properties.

We next turned our attention to their interfacial electronic properties, which are critical for charge injection in transistor applications. Ultraviolet photoelectron spectroscopy (UPS) was employed to determine the *W_F_* of the MXene electrodes and 2H‐MoTe_2_, as well as the valence band position of the semiconductor. As shown in Figure 2i–k, the *W_F_* values of Ti_3_C_2_T_x_, Nb_2_CT_x_, and Mo_2_CT_x_ were measured to be 4.44, 4.72, and 4.23 eV, respectively, while 2H‐MoTe_2_ exhibits a *W_F_* of 4.40 eV and a valence band maximum (VBM) located 0.33 eV below the Fermi level (*E_F_*). These values, which are consistent with previous reports [14, 24, 44, 45], were used to construct the band alignment diagram in Figure 2l. The diagram reveals that Nb_2_CT_x_ provides the closest energetic match to the 2H‐MoTe_2_ VBM, indicating a favorable interface for hole injection.

Notably, the work function of MXenes is not determined solely by the transition‐metal element. The metal species can influence the baseline electronic structure through metal d‐orbital contributions and metal–carbon bonding, while surface terminations such as –O, –F, and –OH further tune the work function by modifying surface dipoles and charge redistribution. Therefore, the experimentally measured work function reflects the combined contribution of MXene composition and its actual surface chemistry. In this study, Nb_2_CT_x_ exhibits the highest UPS‐measured work function among the three MXenes investigated, leading to the closest energetic alignment with the valence band maximum of 2H‐MoTe_2_ and thus a reduced hole‐injection barrier.

## 3 Device Fabrication and Electrical Performance of MXene/2H‐MoTe_2_ FETs

Motivated by the promising band alignment, we integrated Nb_2_CT_x_ with 2H‐MoTe_2_ to fabricate high‐performance field‐effect transistor (FET) arrays. However, direct deposition of MXenes onto 2H‐MoTe_2_ is not feasible due to the mismatch between the hydrophilic nature of MXenes and the intrinsic hydrophobicity of the semiconductor surface. To bridge this materials incompatibility, we adopted a polymer‐assisted transfer strategy. The fabrication process is illustrated in Figure S8, and detailed steps are provided in the **Methods** section.

**FIGURE 3:**
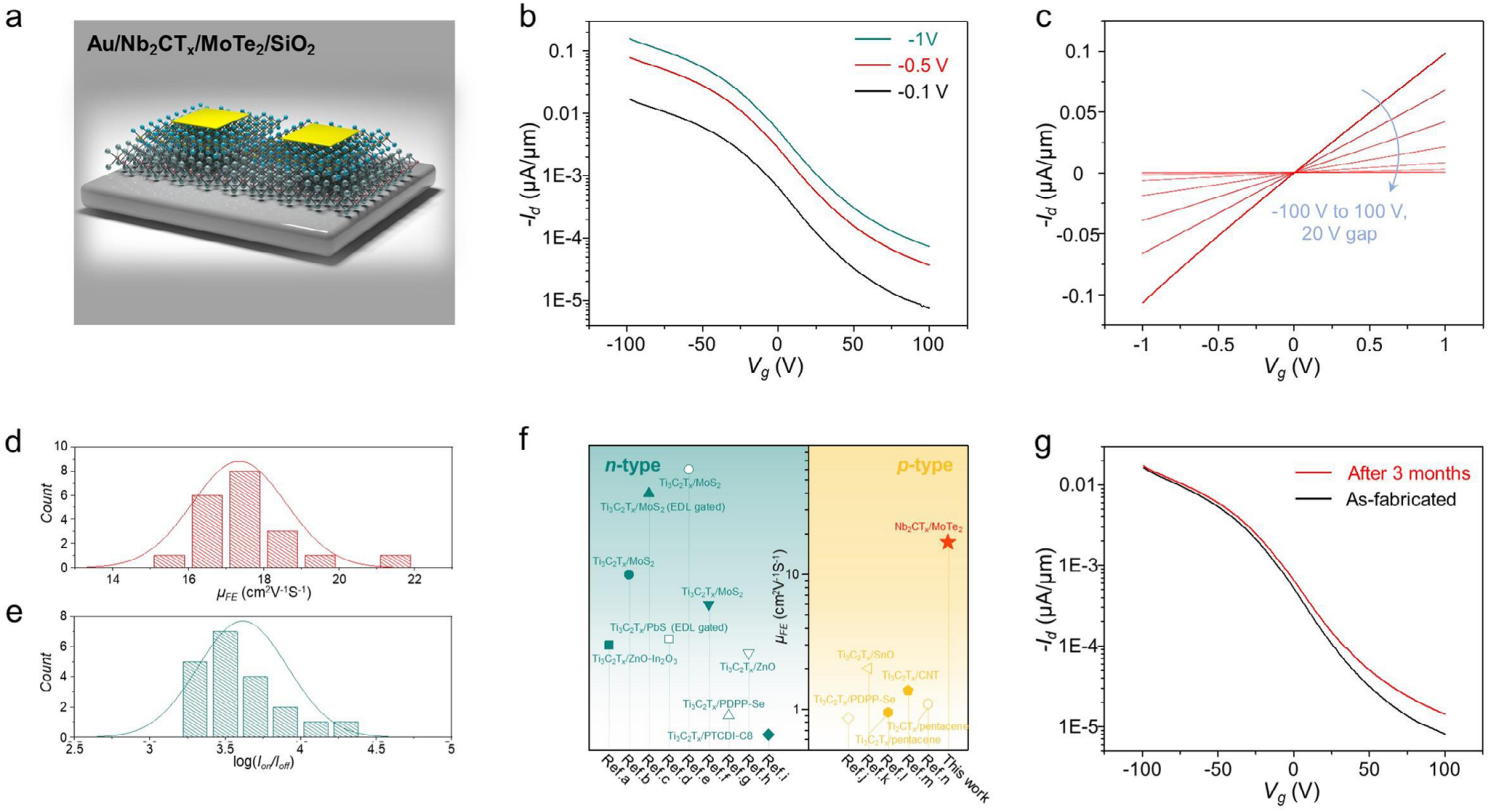
Electrical Characteristics of MXene/2H‐MoTe_2_ FETs. (a) The schematic of the Au/MXene (Nb_2_CT_x_)/2H‐MoTe_2_ device. (b) Transfer curves of a Nb_2_CT_x_/2H‐MoTe_2_ device at different *V_ds_* voltages (−0.1, −0.5, and −1 V). (c) *I_d_‐V_ds_* curves of a Nb_2_CT_x_/2H‐MoTe_2_ device measured under various gate voltages. (d, e) Statistical distributions of (d) effective hole mobility and (e) log (*I_on_*/*I_off_*) of 20 individual transistors at different points on the wafer. (f) Comparison of the field‐effect hole mobility (*µ_FE_*) values with those of the reported MXene‐based transistors. (Open symbols: individual devices; filled symbols: device arrays) (reference: a, [46] b, [47] c, [35] d, [12] e, [48] f, [14] g, [10] h, [9] i, [11] j, [10] k, [9] l, [11] m, [49], and n [50]) (g) Transfer curves of the Nb_2_CT_x_/2H‐MoTe_2_ device as‐fabricated and after 3 months in dry air.

As shown in Figure 3a, the final device structure comprises patterned Au/MXene source/drain electrodes laminated onto the 2H‐MoTe_2_ film. A 20 nm‐thick Au capping layer was pre‐deposited on the MXene to improve lateral conductivity and protect the underlying interface. Although MXene film thickness and stacking may influence contact resistance, all MXene electrodes in this study were prepared using the same spray‐coating protocol with a controlled thickness. The 20 nm‐thick Au capping layer was introduced to improve lateral conductivity and reduce possible variations in electrode series resistance. Therefore, while thickness‐related effects cannot be fully excluded, the consistent electrode preparation allows a reasonable comparison among different MXene compositions.

With this configuration established, we then evaluated the electrical performance of the devices. The corresponding optical images of the 2H‐MoTe_2_ film and the completed device are presented in Figure S9. The electrical performance of the fabricated devices was systematically evaluated under ambient conditions. To determine the optimal MXene contact material in this work, we first compared the electrical performance of 2H‐MoTe_2_ transistors with Ti_3_C_2_T_x_, Nb_2_CT_x_, and Mo_2_CT_x_ electrodes (Figure S10). Among these, devices with Nb_2_CT_x_ contacts exhibited the best transfer characteristics, with a high hole mobility of ∼19.8 cm^2^ V^−1^s^−1^. Moreover, they also showed the lowest contact resistance (Figure S11), in good agreement with the trends observed in the UPS‐derived work function alignment. Based on these results, we selected Nb_2_CT_x_ for all subsequent device studies and conducted a more in‐depth investigation of its performance in 2H‐MoTe_2_‐based transistors. This trend indicates that the experimentally measured work function provides a useful guide for screening MXene contacts, although the final device performance also depends on film quality, interface cleanliness, and possible Fermi‐level pinning.

The electrical performance of a representative device with a channel width/length ratio (W/L) of 100 µm/50 µm is shown in Figure 3b,c. The transfer curves measured at different drain voltages (e.g., −0.1, −0.5, and −1 V) clearly show *p*‐type transistor behavior, with a high hole mobility of ∼19.8 cm^2^ V^−1^s^−1^ and an on/off current ratio of ∼5.23 × 10^4^. The output characteristics (Figure 3c) confirm the excellent contact between Nb_2_CT_x_ and 2H‐MoTe_2_, exhibiting linear and symmetric *I_DS–_V_DS_* curves under varying gate voltages. To evaluate device uniformity, 20 devices with identical geometry were randomly selected and measured across the substrate. As shown in Figure 3d,e and Figure S12, the performance distribution is narrow, with average hole mobility *µ* = 17.4 ± 1.2 cm^2^ V^−1^s^−1^ and average log(*I_on_/I_off_*) = 3.6 ± 0.3. The good uniformity is attributed to the favorable interfacial band alignment and the mild lamination process that avoids interface damage, as further confirmed by cross‐sectional STEM analysis (Figure S13), which reveals a sharp and well‐defined 2H‐MoTe_2_ interface.

Although conventional metals such as Pt, Pd, and Au possess work functions favorable for *p*‐type 2D semiconductors, their high melting points (>1000°C) necessitate high‐energy deposition processes, which can damage the semiconductor interface and degrade contact quality [26, 51]. To highlight the advantages of our approach, we compared Nb_2_CT_x_/2H‐MoTe_2_ devices with Pt‐contacted counterparts. As shown in Figure S14, Nb_2_CT_x_‐based devices exhibit significantly superior performance. Moreover, when benchmarked against previously reported 2H‐MoTe_2_ (via CVD) transistors, our devices achieve markedly higher *I_on_/I_off_* and enhanced mobility (Figure S15 and Table S1) [26, 27, 28, 29, 30, 31, 32, 33]. Furthermore, as summarized in Figure 3f, our devices also outperform nearly all previously reported MXene‐based transistors in terms of mobility. To the best of our knowledge, this work represents the first demonstration of wafer‐scale MXene/2D semiconductor contact engineering in *p*‐type systems, and the first incorporation of Nb_2_CT_x_ as an effective contact material. These results underscore the significant potential of Nb_2_CT_x_ for high‐performance *p*‐type 2D electronics. In addition to excellent electrical performance, the devices also exhibited outstanding stability in dry air, with only negligible degradation observed after 3 months (Figure 3g).

It is worth noting that the choice of transfer polymer plays a critical role in device performance. Compared with PMMA, the devices fabricated using a cellulose acetate (CA)‐assisted transfer method exhibited significantly degraded characteristics (Figure S16), likely due to unintentional doping introduced during the transfer process [52]. Such sensitivity to transfer chemistry is commonly observed in TMD‐based devices and underscores the importance of polymer selection to achieve clean and reproducible semiconductor interfaces. These results demonstrate that Nb_2_CT_x_ is a highly promising contact material for high‐performance *p*‐type 2D transistors, offering low contact resistance, excellent stability, and scalable fabrication compatibility.

## 4 Temperature‐Dependent Contact Behavior of Nb_2_CT_x_/2H‐MoTe_2_ Devices

**FIGURE 4:**
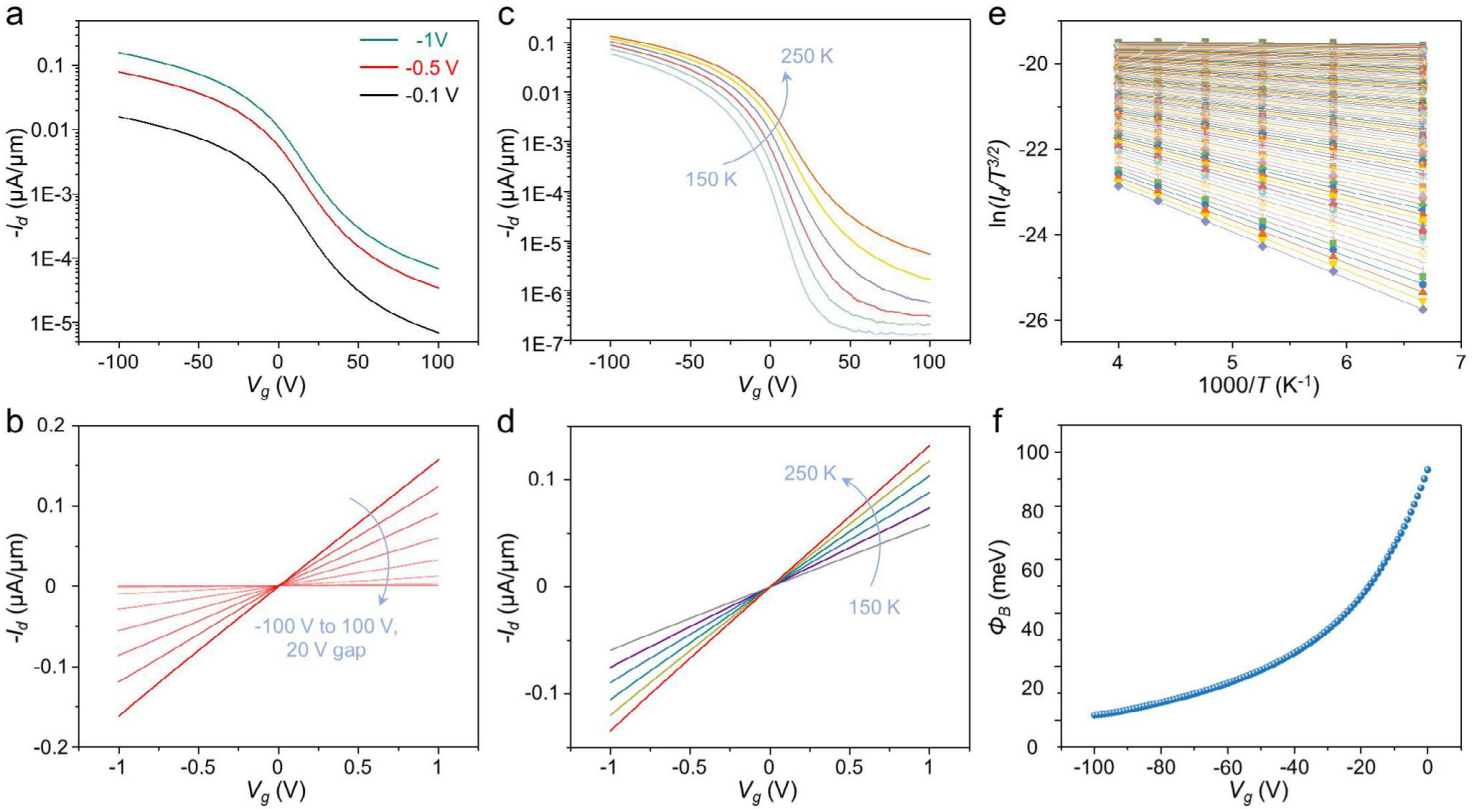
Temperature‐Dependent Electrical Characteristics of MXene/2H‐MoTe_2_ FETs. (a) Transfer curves of a Nb_2_CT_x_/2H‐MoTe_2_ device at different *V_ds_* voltages (−0.1, −0.5, and −1 V) in vacuum. (b) *I_d_‐V_ds_* curves of a Nb_2_CT_x_/2H‐MoTe_2_ device measured under various gate voltages in vacuum. (c) The temperature‐dependent *I_d–_V_g_* transfer curves of the Nb_2_CT_x_/2H‐MoTe_2_ device. (d) The temperature‐dependent *I_d_‐V_ds_* curves of a Nb_2_CT_x_/2H‐MoTe_2_ (*V_g_* = −100 V). (e) Richardson plot, ln(*I_d_/T^3/2^*) vs. 1000/T at different gate voltages; the slope of the lines yields Schottky barrier (*Φ_B_*) as a function of gate voltage. (f) The gate voltage‐dependent extracted effective *Φ_B_*.

To gain deeper insight into the contact properties between Nb_2_CT_x_ and 2H‐MoTe_2_, temperature‐dependent electrical measurements were conducted under vacuum conditions from 150 to 250 K (Figure 4). Figure 4a displays representative transfer curves for a Nb_2_CT_x_/2H‐MoTe_2_ device in vacuum at different *V_DS_* voltages (−0.1, −0.5, and −1 V). The corresponding output characteristics presented in Figure 4b display linear behavior, confirming low‐resistance contacts. We then observed a notable increase in the *I_on_/I_off_* ratio as the temperature decreased, reaching values up to ∼10^5^ at 150 K (Figure 4c). This enhancement is primarily attributed to the suppression of thermionic‐assisted carrier emission at lower temperatures, which effectively reduces the off‐state current. The output characteristics at various temperatures (Figure 4d) further display linear *I–V* behavior, indicating minimal Schottky barriers at the Nb_2_CT_x_/2H‐MoTe_2_ interface.

Moreover, to quantitatively assess the contact properties of the device, we extracted the Schottky barrier height (*Φ_B_*) by analyzing the activation energy. In a Schottky‐barrier field‐effect transistor, the reverse‐biased contact typically dominates the voltage drop across the device and largely determines its overall electrical behavior. The current density arising from thermionic emission over the metal–semiconductor junction under reverse‐bias conditions can be described by the following equation [53]:

J=A∗Tαexp[−qΦB/κbT][1−exp(−qV/κbT)]

where *A^∗^* is the Richardson constant, *V* is the applied voltage, *T* is the temperature, *α* is an exponent equal to 2 for bulk semiconductors and to 3/2 for 2D semiconductors, and *𝜅b* is Boltzmann's constant.

Using this equation, the Schottky barrier height (*Φ_B_*) was determined from the slope of the Richardson plot, ln(*I_ds_*/T^3/2^) vs. 1000/T (Figure 4e), as a function of gate voltage (Figure 4f). At large negative gate voltages, *Φ_B_* deviates from a linear trend and approaches a small value on the order of a few meV, indicating the onset of tunneling‐assisted carrier injection [53]. This reduction in the effective barrier height corresponds to a lower contact resistance under strong negative gate bias, which aligns with the small contact resistance values obtained from the transfer length method at high turn‐on gate voltages.

## 5 Conclusions

This study systematically demonstrates the critical role of compositional engineering in optimizing MXene‐based contacts for *p*‐type 2D transistors. By extending the scope of MXene electrodes beyond the conventional Ti_3_C_2_T_x_, we have identified Nb_2_CT_x_ as the best‐performing contact material for 2H‐MoTe_2_ among the MXenes evaluated in this study. Its enhanced performance —manifested in high hole mobility (∼17 cm^2^ V^−^^1^ s^−^^1^), large on/off ratios (>10^3^), and excellent device uniformity—is attributed to its ideal work function alignment, which enables the formation of clean, low‐resistance interfaces. This favorable band alignment is further validated by temperature‐dependent measurements, which reveal a remarkably low Schottky barrier height. Our results not only establish Nb_2_CT_x_ as a scalable and high‐performance alternative to conventional high‐work‐function metals but also unlock the vast compositional space of MXenes for contact engineering. More broadly, other MXenes, such as V_2_CT_x_, Ta_2_CT_x_, Ta_4_C_3_T_x_, and the investigated MXenes with different terminations, may also serve as promising *p*‐type contact candidates if suitable work functions, stable surface terminations, and clean interfaces can be achieved. Future studies using MXenes with more precisely controlled surface terminations would be valuable for establishing a quantitative composition–termination–work‐function–contact‐property relationship. This work provides a clear pathway toward next‐generation, solution‐processable *p*‐type 2D electronic and optoelectronic devices.

## Author Contributions

H.N.A. and X.Z. conceived this project. T.G. and M.C. designed most of the experimental work, including the MXene and semiconductor synthesis and device fabrication. T.G., M.C., and X.Z. characterized the quality of MXenes and MoTe_2_. Y.W. D.Z., and N.C. fabricated the MXene films. C.L. performed the TEM and STEM measurements. T.G., X.X., and M.C. conducted the transistors. T.G., L.L., and T.D.A. conducted the measurement of transistors. T.G. and A.C. discussed the results and wrote the manuscript. All authors discussed the results and contributed to the writing of the manuscript.

## Conflicts of Interest

The authors declare no conflicts of interest.

## Supporting information




**Supporting File**: advs76591‐sup‐0001‐SuppMat.docx.

## Data Availability

The data that support the findings of this study are available from the corresponding author upon reasonable request.
