# Supplementary material for: MXenes Contacts for p‐type 2D Electronics
Source: Adv Sci (Weinh). 2026 Aug 3:e76591. Online ahead of print. doi: 10.1002/advs.76591 (PMC13430937; doi:10.1002/advs.76591)
Supplement: Supplementary file 1 — Supporting File: advs76591‐sup‐0001‐SuppMat.docx. [file ADVS-9999-e76591-s001.docx]

Supplementary information for

**MXenes Contacts for *p*-type 2D Electronics**

Tianchao Guo^1†^, Maolin Chen^1†^, Yizhou Wang^1,2^, Chen Liu^1^, Xiangming Xu^3^, Linqu Luo^1^, Dekang Zhu^1^, Ning Chu^1^, Xiaowen Zhang^1^, Alfonso Caraveo^1^, Thomas D. Anthopoulos^4^, Xixiang Zhang^1^*, Husam N. Alshareef^1,2^*

^1^Materials Science and Engineering, Physical Science and Engineering Division, King Abdullah University of Science and Technology (KAUST), Thuwal 23955-6900, Saudi Arabia

^2^Center for Renewable Energy and Storage Technology (CREST), King Abdullah University of Science and Technology (KAUST), Thuwal 23955-6900, Saudi Arabia

^3^Shanghai Institute of Microsystem and Information Technology, Chinese Academy of Sciences, Shanghai 20050, China

^4^Photon Science Institute, Henry Royce Institute, Department of Electrical and Electronic Engineering, The University of Manchester, Manchester M13 9PL, United Kingdom

^†^These authors contributed equally.

*Corresponding author.

E-mail: xixiang.zhang@kaust.edu.sa; husam.alshareef@kaust.edu.sa

Keywords: MXenes, solution-processed, *p*-type transistors, 2D materials

**Methods**

*Synthesis of MXenes*

Ti_3_C_2_T_x_, Nb_2_CT_x_, and Mo_2_CT_x_ MXenes were synthesized by selectively etching their MAX precursors, followed by intercalation and exfoliation.^[1]^ For Ti_3_C_2_T_x_, 1 g of Ti_3_AlC_2_ powder (400 mesh, Lanzhou Kai Kai Ceramic Materials Co. Ltd) was slowly added to a solution containing of 1 ml 49% HF, 6 ml 12 M HCl, and 3 ml deionized (DI) water, and stirred at 42 °C for 15 h. The etched product was washed with DI water until the pH reached ~6. To achieve delamination, 20 ml of 0.75 M LiCl solution was added to the sediment, stirred for 180 min, and washed by six centrifugation cycles. A few-layer MXene suspension was finally collected by centrifugation at 3500 rpm for 30 min. For Nb_2_CT_x_ and Mo_2_CT_x_, 1 g of Nb_2_AlC or 2 g Mo_2_Ga_2_C powder (400 mesh, Lanzhou Kai Kai Ceramic Materials Co. Ltd) was etched in 20 ml of 49% HF at 55 °C for 48 h (Nb_2_AlC) or 42 °C for 7 days (Mo_2_Ga_2_C), respectively. After washing to neutral pH, intercalation was performed using 20 ml of 5 wt% tetramethylammonium hydroxide (TMAOH) under stirring at 400 rpm for 15 h. After repeated washing, the resulting few-layer MXene suspensions were obtained by centrifugation at 3500 rpm for 30 min.

*Growth of the MoTe_2_ film*

MoTe_2_ films (8-9 nm) were prepared via tellurization of pre-deposited Mo layers under atmospheric pressure in a tube furnace equipped with mass flow controllers and a vacuum pump.^[2]^ Mo layers were first deposited on 300 nm-SiO_2_/Si substrates at room temperature using a Singulus ROTARIS magnetron sputtering system. The deposition was carried out at 298 K in an Ar atmosphere (~3×10^-3^ mbar) using a 4-inch Mo target with 500 W DC power, with substrates positioned ~10 cm from the target. For tellurization, the substrates were placed face-down on an alumina boat containing Te powder was placed at the center of the heating zone in a one-inch quartz tube. After evacuating the quartz tube to <10 Pa, Ar was introduced until atmospheric pressure was reached, followed by flowing Ar (4 sccm) and H_2_ (5 sccm). The furnace was ramped to 650 °C in 20 min, held for 30 min, and then cooled to room temperature naturally.

*Device fabrication*

MXene films (Ti_3_C_2_T_x_, Nb_2_CT_x_, and Mo_2_CT_x_) (~20 nm) were first deposited on separate SiO_2_/Si substrates by spray coating. Prior to deposition, the SiO_2_ surface was treated with UV–ozone to improve hydrophilicity. A 20 nm-thick Au capping layer was then thermally evaporated onto the MXenes to enhance electrical conductivity and environmental stability. Standard photolithography and dry etching were used to pattern the Au/MXene electrodes. To transfer the patterned electrodes, a PMMA support layer was spin-coated and baked at 180 °C, followed by immersion in dilute buffered oxide etchant (BOE) to release the PMMA/Au/MXene stack. After rinsing in deionized water, the stack was transferred onto the pre-patterned 2H-MoTe_2_ substrate. Finally, the PMMA was removed, completing the fabrication of bottom-gated FETs.

*Characterization*

The morphologies of Ti_3_AlC_2_, Nb_2_AlC, and Mo_2_Ga_2_C, were characterized using scanning electron microscopy (SEM, Zeiss Merlin, Carl Zeiss SMT AG). The morphologies of Ti_3_C_2_T_x_, Nb_2_CT_x_, and Mo_2_CT_x_ were characterized using atomic force microscopy (AFM, Bruker Dimension Icon SPM). X-ray diffraction (XRD) patterns were recorded using a Bruker D8 Advance diffractometer with Cu Kα radiation (λ = 1.5406 Å) to confirm phase purity. Raman spectra were obtained with a Wintec Apyron Raman spectrometer using a 532 nm excitation laser. The transmission electron microscopy (TEM) sample was prepared using a focused ion beam (FIB, Helios G4, FEI), and TEM imaging was performed on a Titan G2 60-300 (FEI) equipped with a spherical aberration corrector, operated at 300 kV. X-ray photoelectron spectroscopy (XPS) was carried out on a Thermo Scientific ESCALAB 250Xi system to analyze the surface chemical states, and ultraviolet photoelectron spectroscopy with a He–I excitation (21.22 eV) source was used to determine work functions and valence band positions. Electrical measurements of MXene/2H-MoTe_2_ transistors were conducted using an Agilent B1500A semiconductor device analyzer connected to a Summit-11600 AP (Cascade Microtech) probe station under ambient conditions. Temperature-dependent transport characteristics were measured in a cryogenic probe station over the range of 150–250 K using a source-meter (Keysight B2912A).


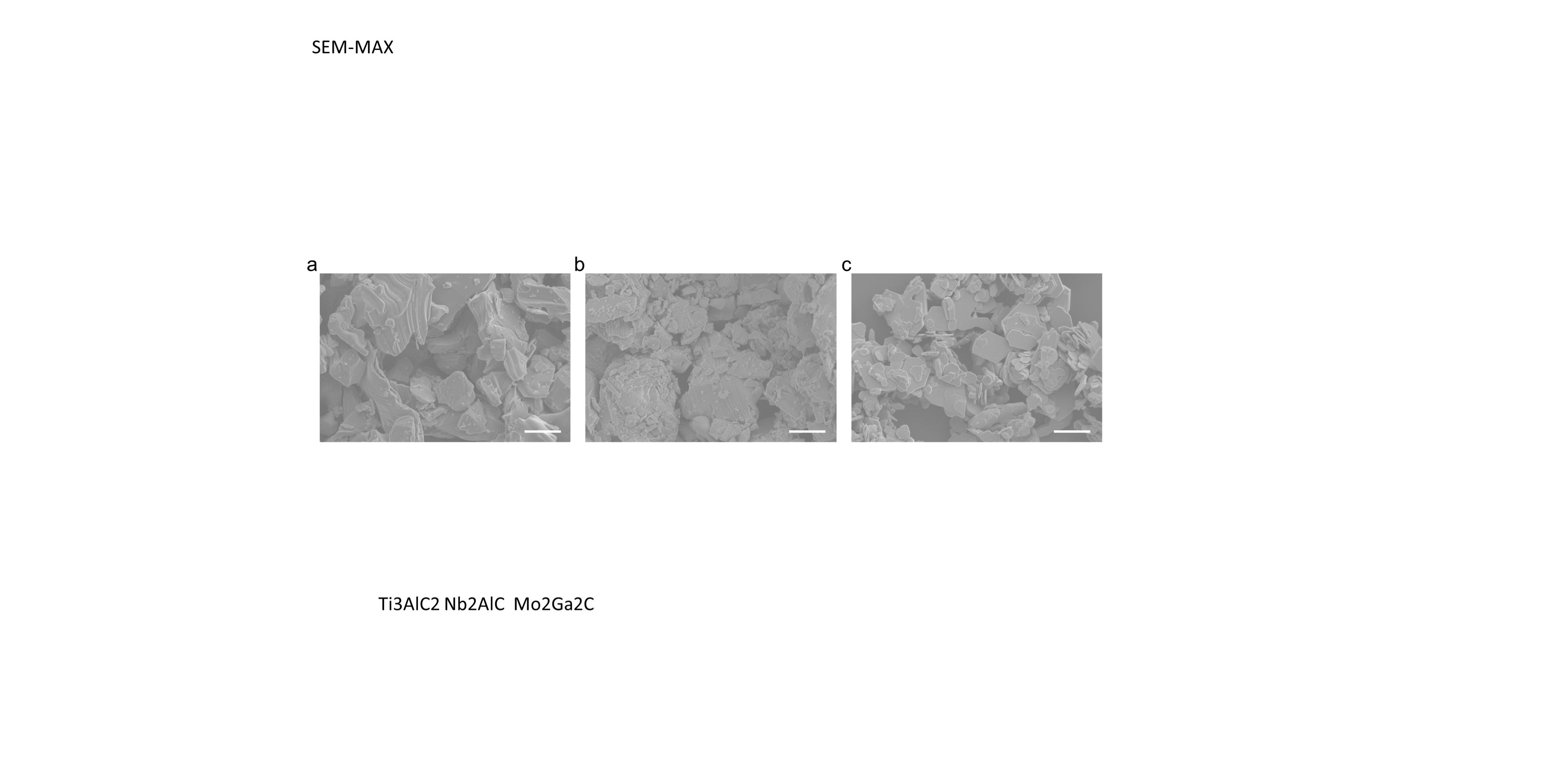


Figure S1. SEM images of a) Ti_3_AlC_2_, b) Nb_2_AlC, and c) Mo_2_Ga_2_C. Scale bar, 4 μm.


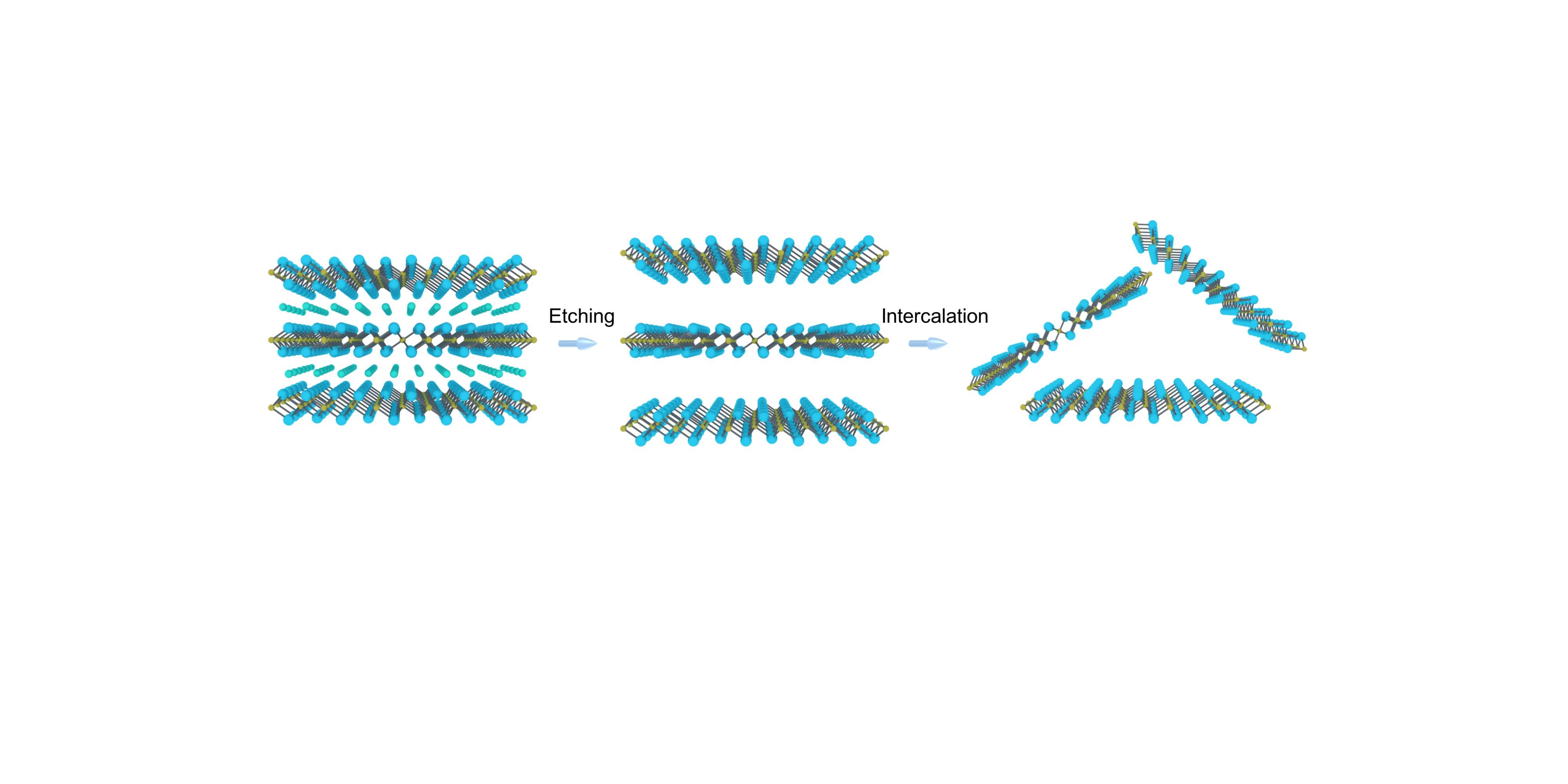


Figure S2. Schematic illustration of MXenes synthesis process.


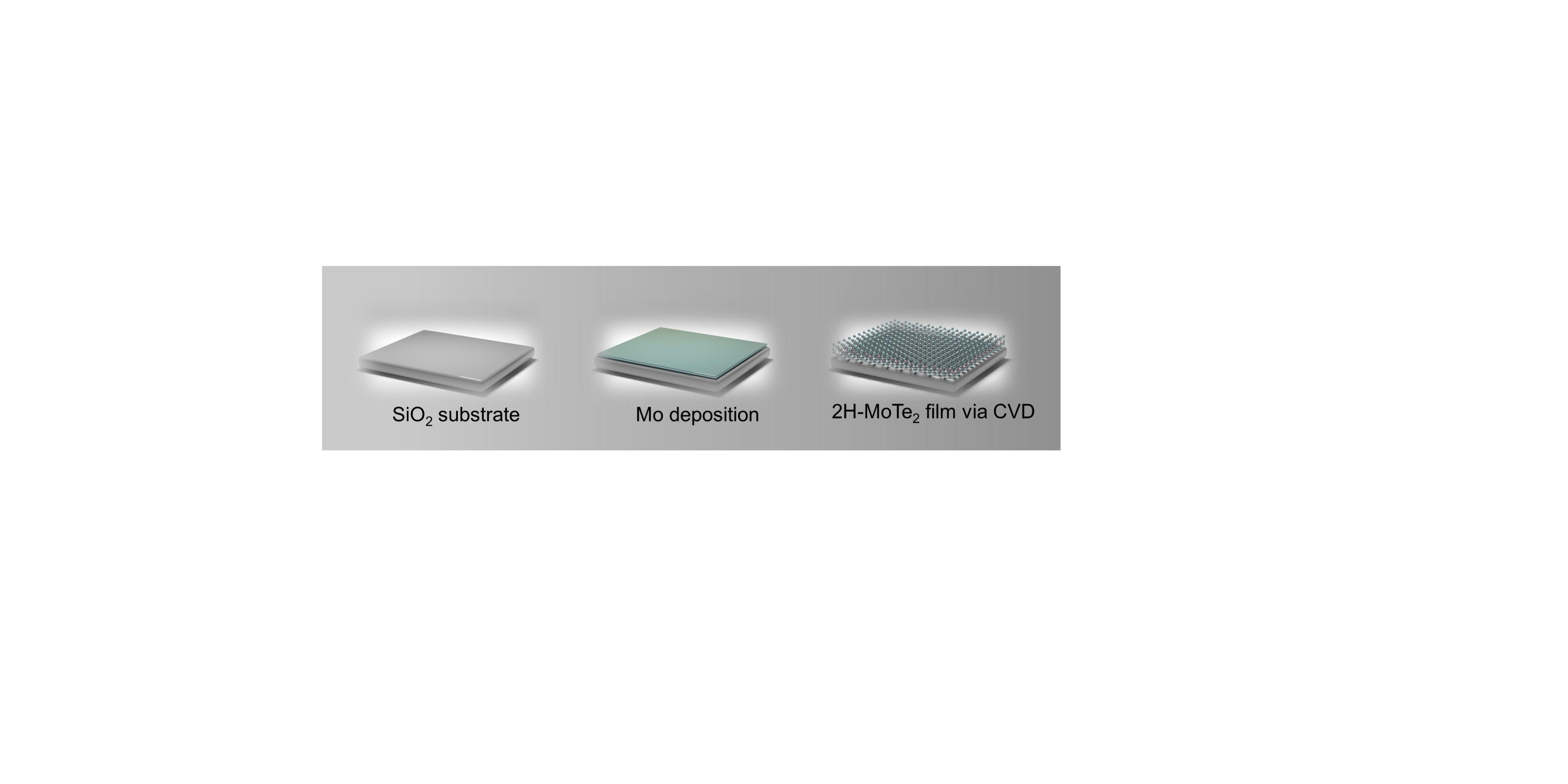


Figure S3. Schematic illustration of 2H-MoTe_2_ synthesis process.


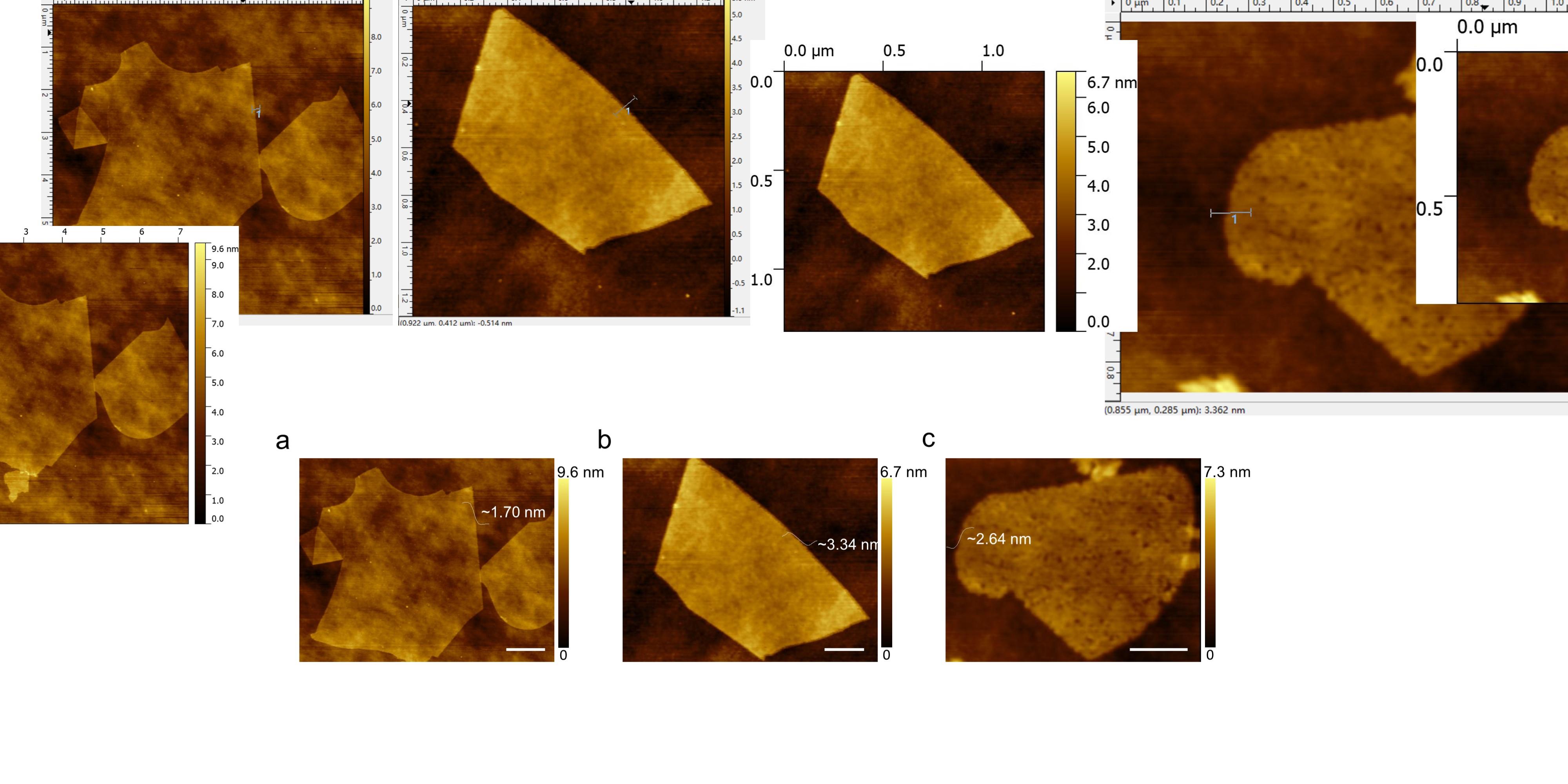


Figure S4. AFM images of a) Ti_3_C_2_T_x_, b) Nb_2_CT_x_, and c) Mo_2_CT_x_. Scale bars are a) 1 μm, b) 200 nm, and c) 200 nm.


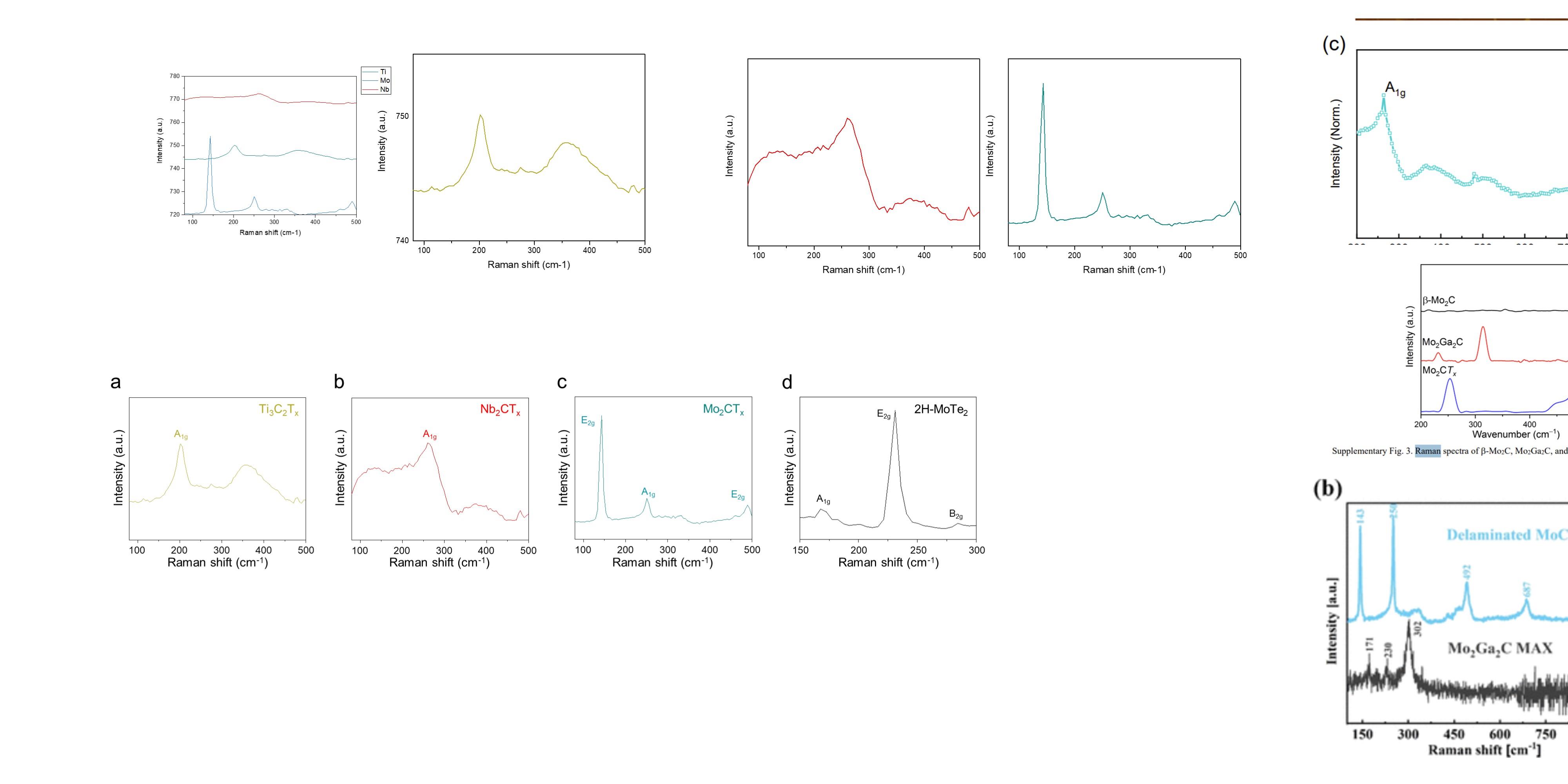


Figure S5. Raman spectra of a) Ti_3_C_2_T_x_, b) Nb_2_CT_x_, c) Mo_2_CT_x_, and d) 2H-MoTe_2_.


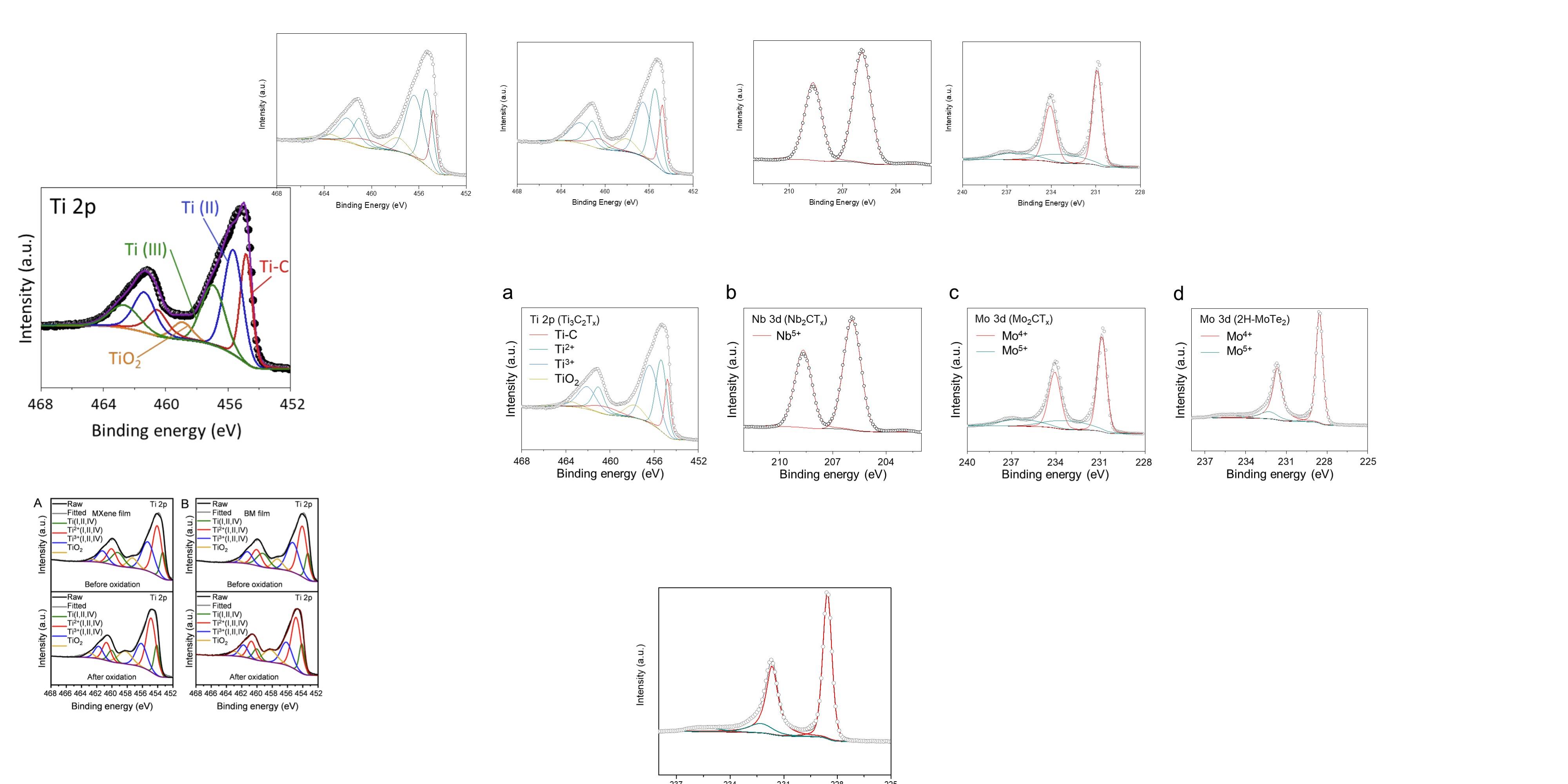


Figure S6. XPS spectra of a) Ti_3_C_2_T_x_, b) Nb_2_CT_x_, c) Mo_2_CT_x_, and d) 2H-MoTe_2_.


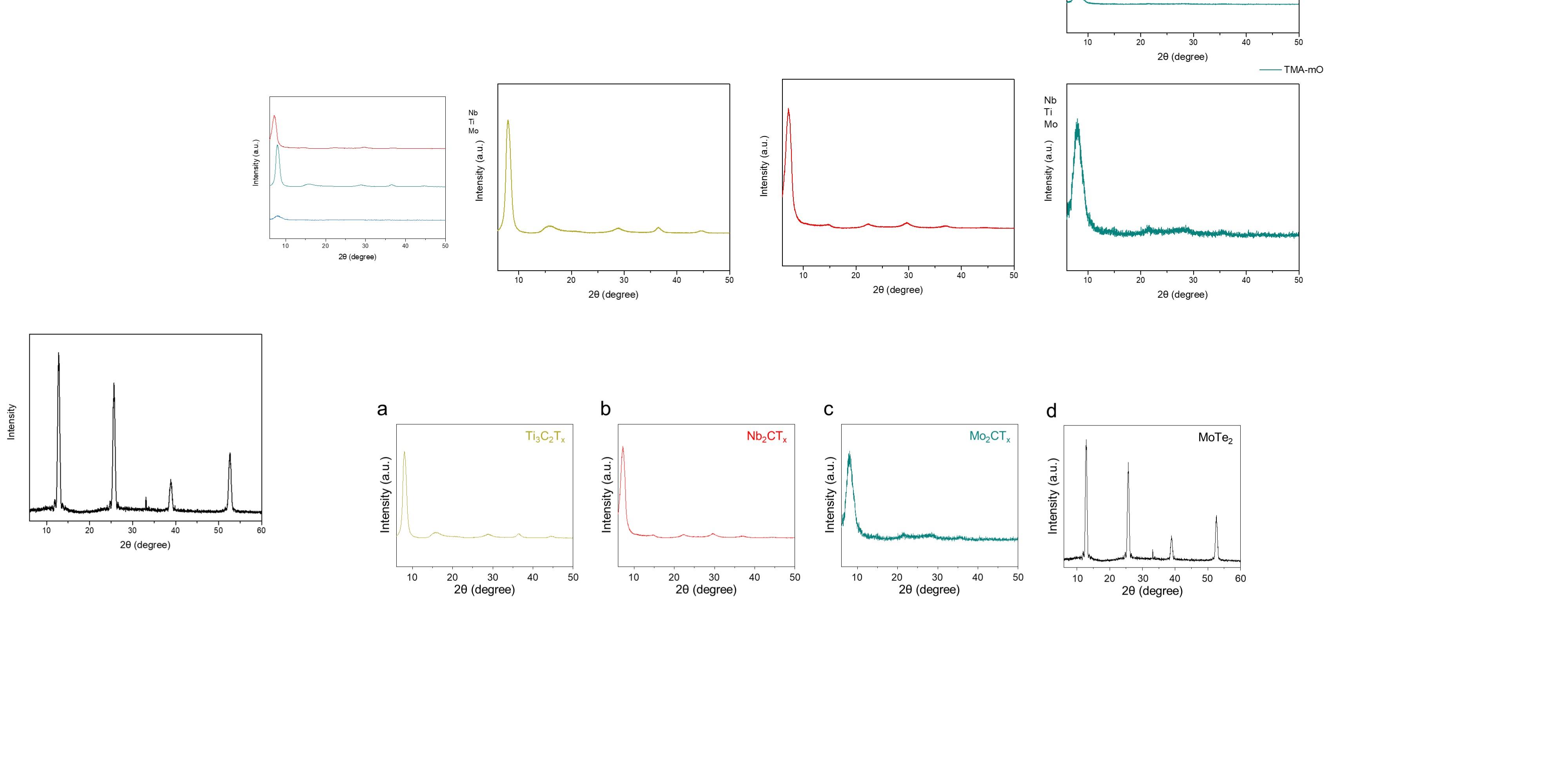


Figure S7. XRD patterns of a) Ti_3_C_2_T_x_, b) Nb_2_CT_x_, c) Mo_2_CT_x_, and d) 2H-MoTe_2_.


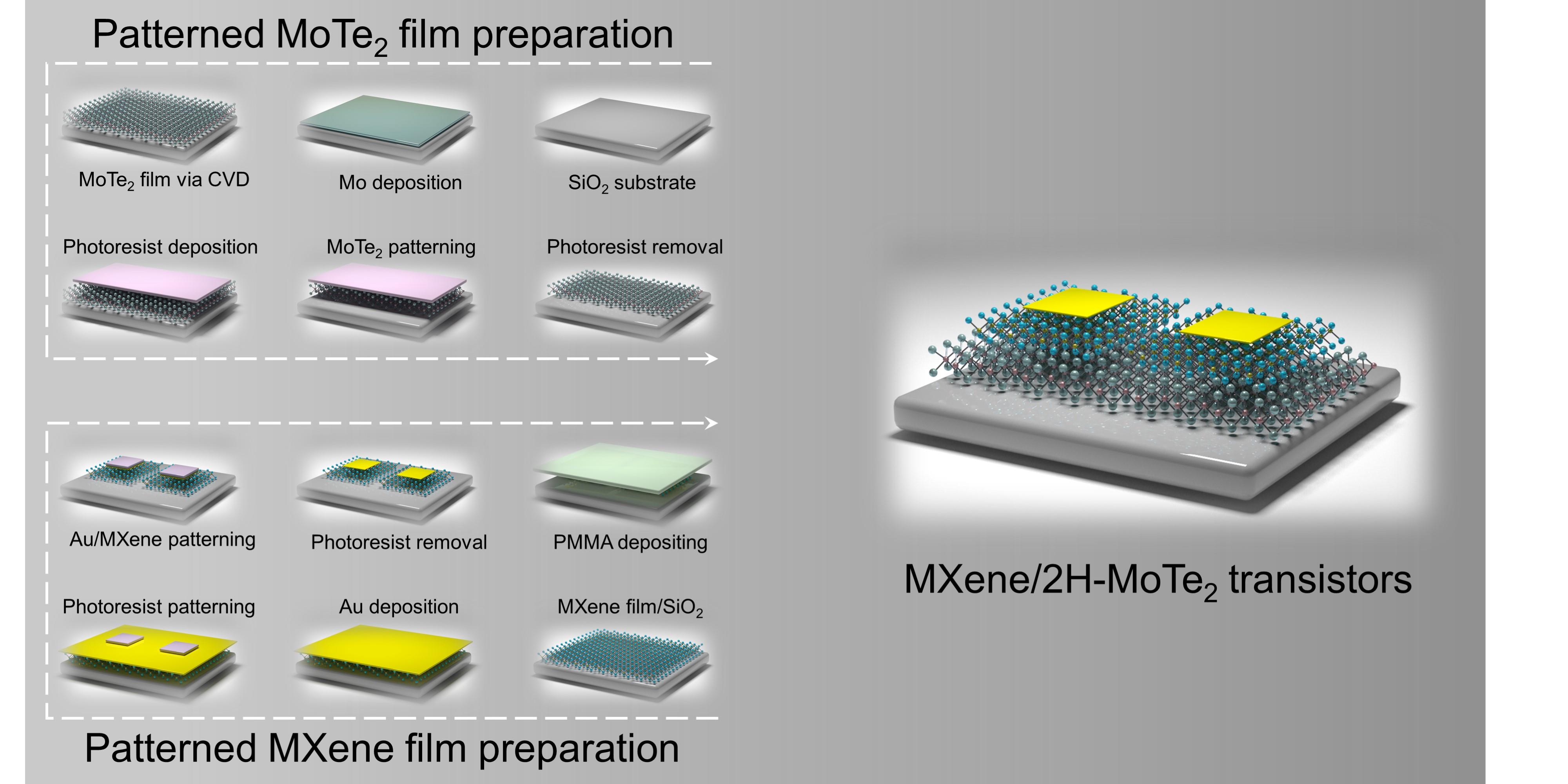


Figure S8. Schematic illustration of the fabrication process for patterned MXene/2H-MoTe_2_ transistors.


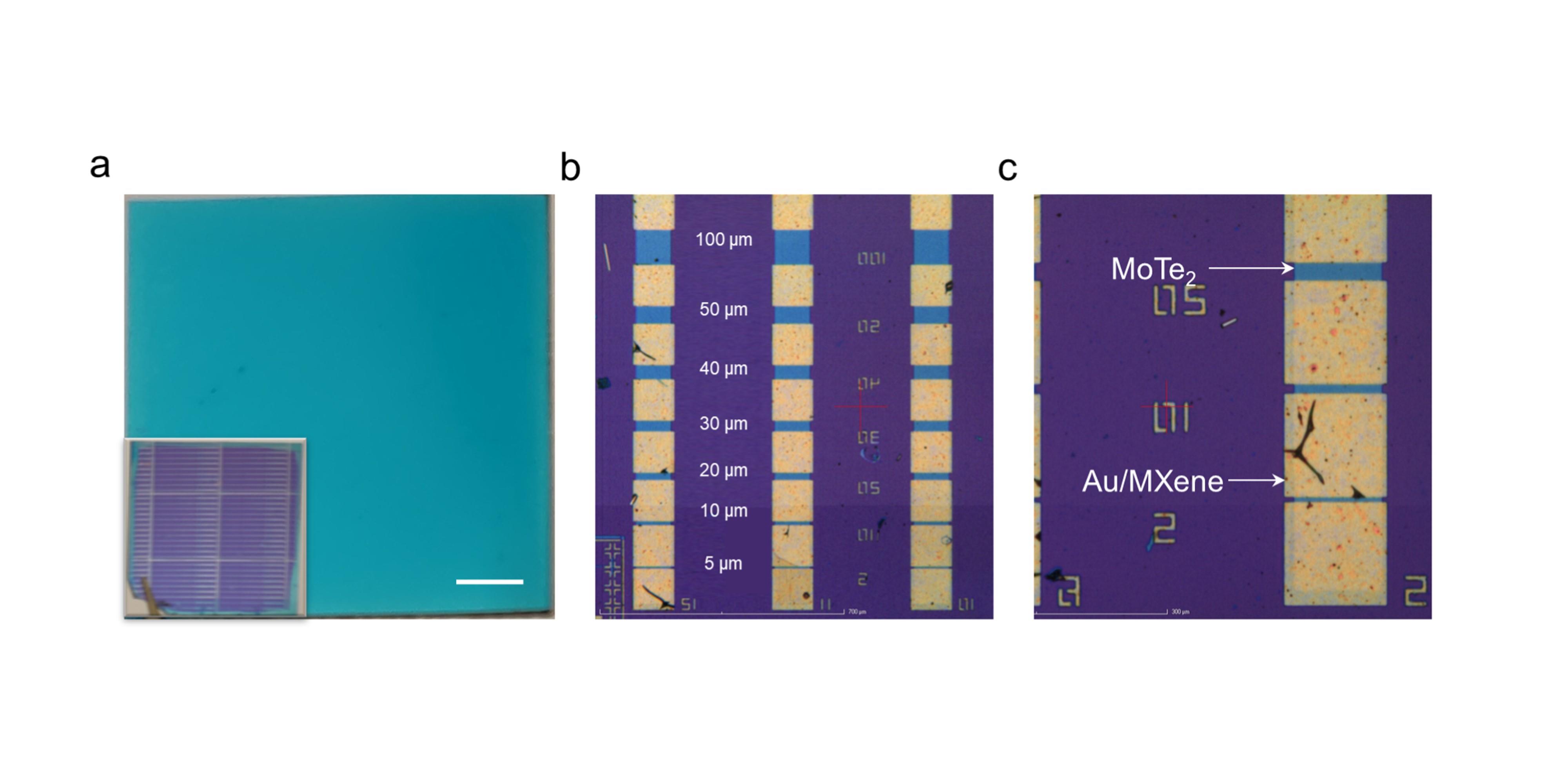


Figure S9. a) Optical image of the synthesized 2H-MoTe_2_ film, scale bar, 2 mm. (Inset: optical image of the fabricated devices). b-c) Optical microscopy image of MXene/2H-MoTe_2_ devices.


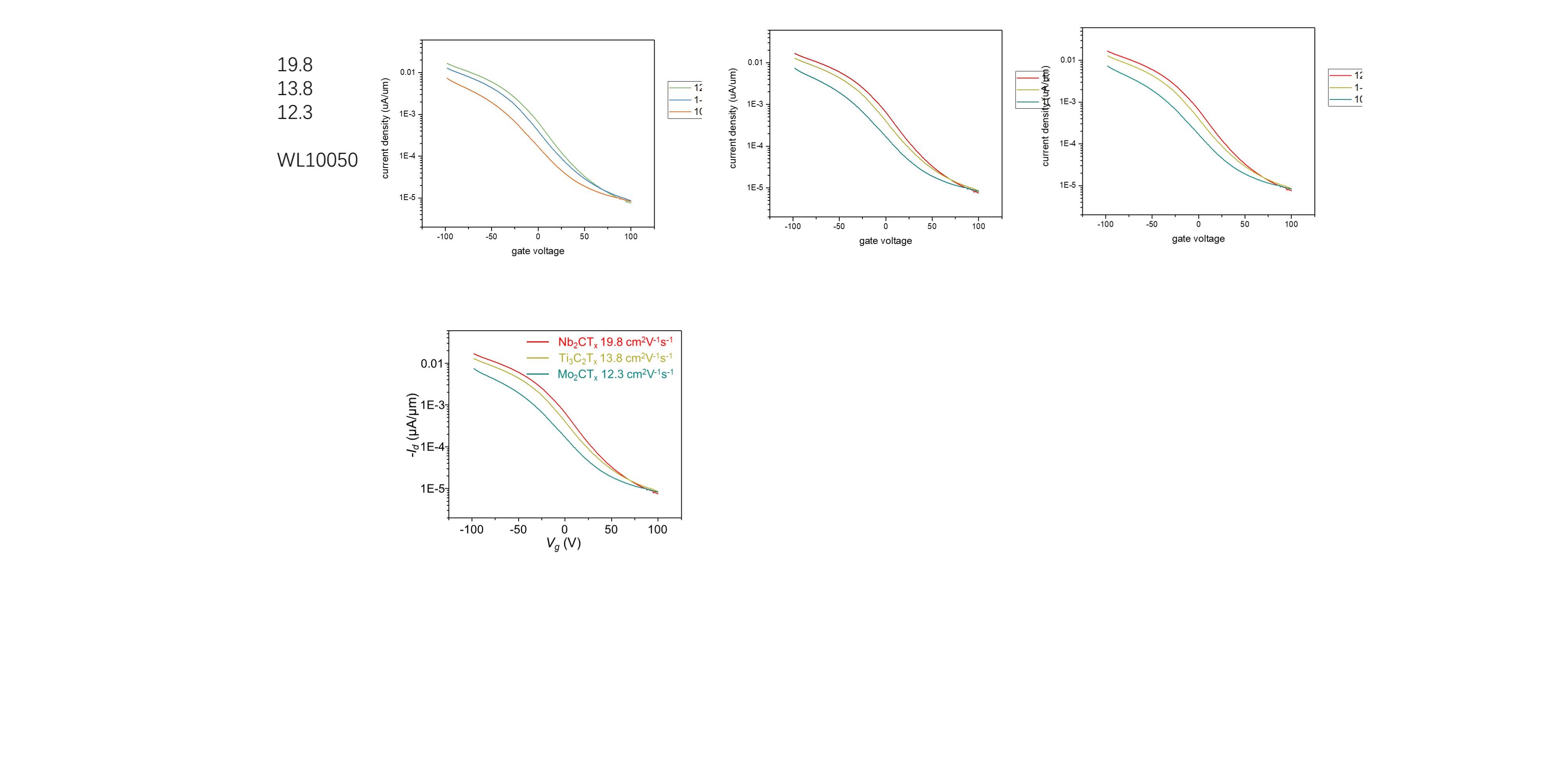


Figure S10. Comparison of transfer characteristics of 2H-MoTe_2_ transistors with different MXene contacts.


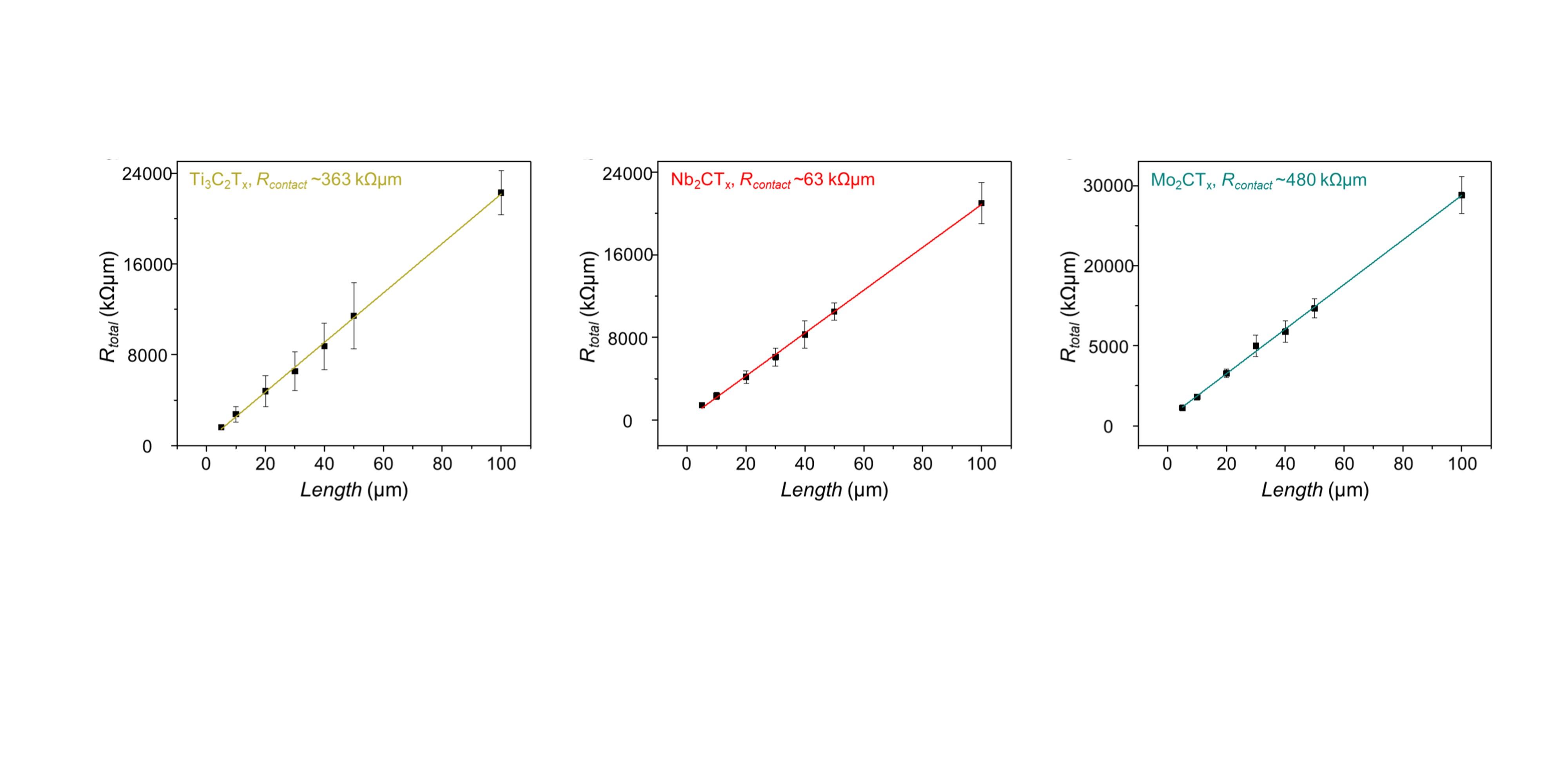


Figure S11. Comparison of contact resistance of 2H-MoTe_2_ transistors with different MXene contacts.


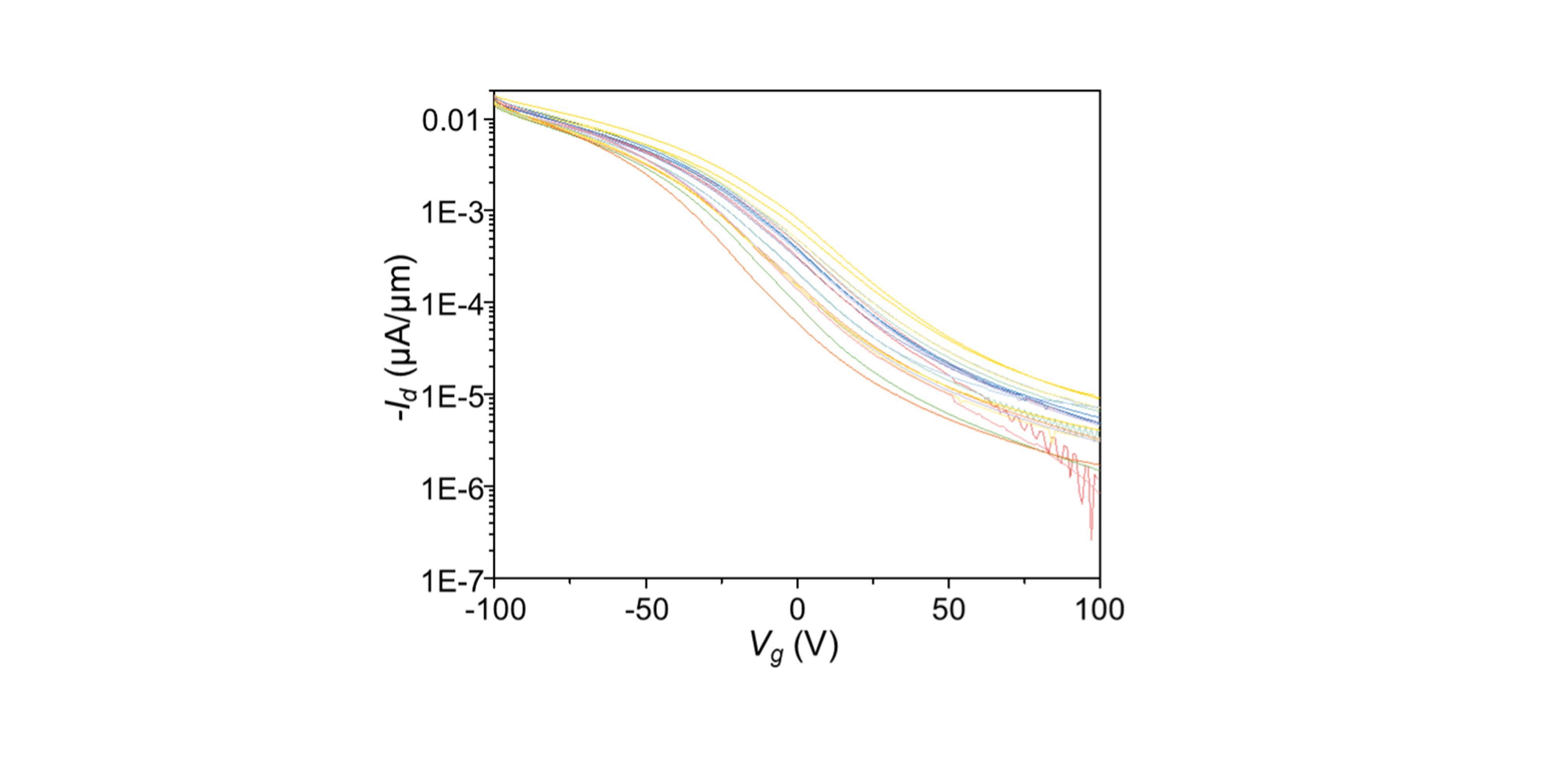


Figure S12. Transfer curves of 2H-MoTe_2_ transistors with Nb_2_CT_x_ contacts.


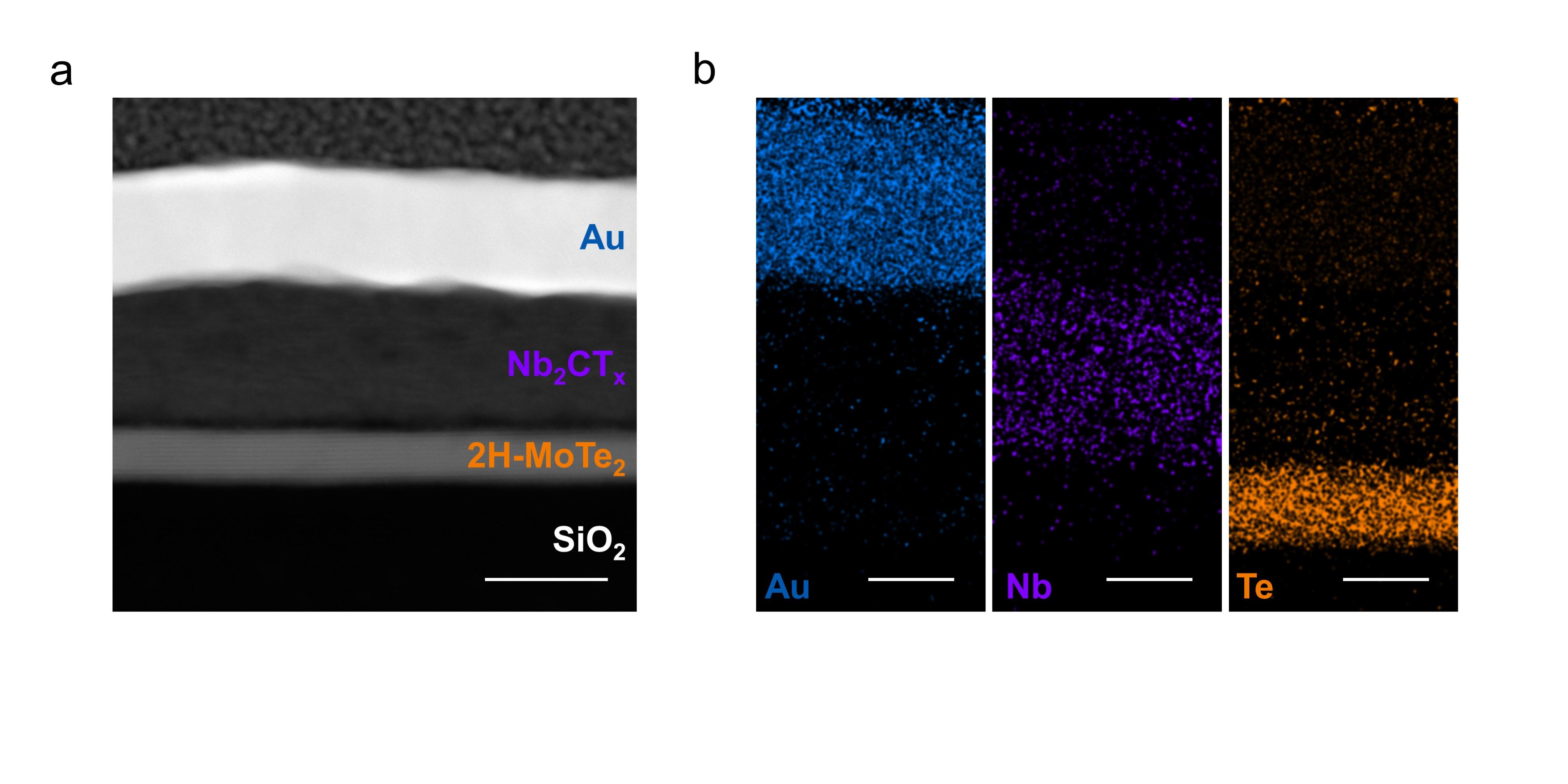


Figure S13. a) High-resolution cross-sectional STEM image of Nb_2_CT_x_/2H-MoTe_2_/SiO_2_. b) EDS elemental mapping for Au, Nb, and Te. Scale bars are a) 20 nm and b)10 nm.


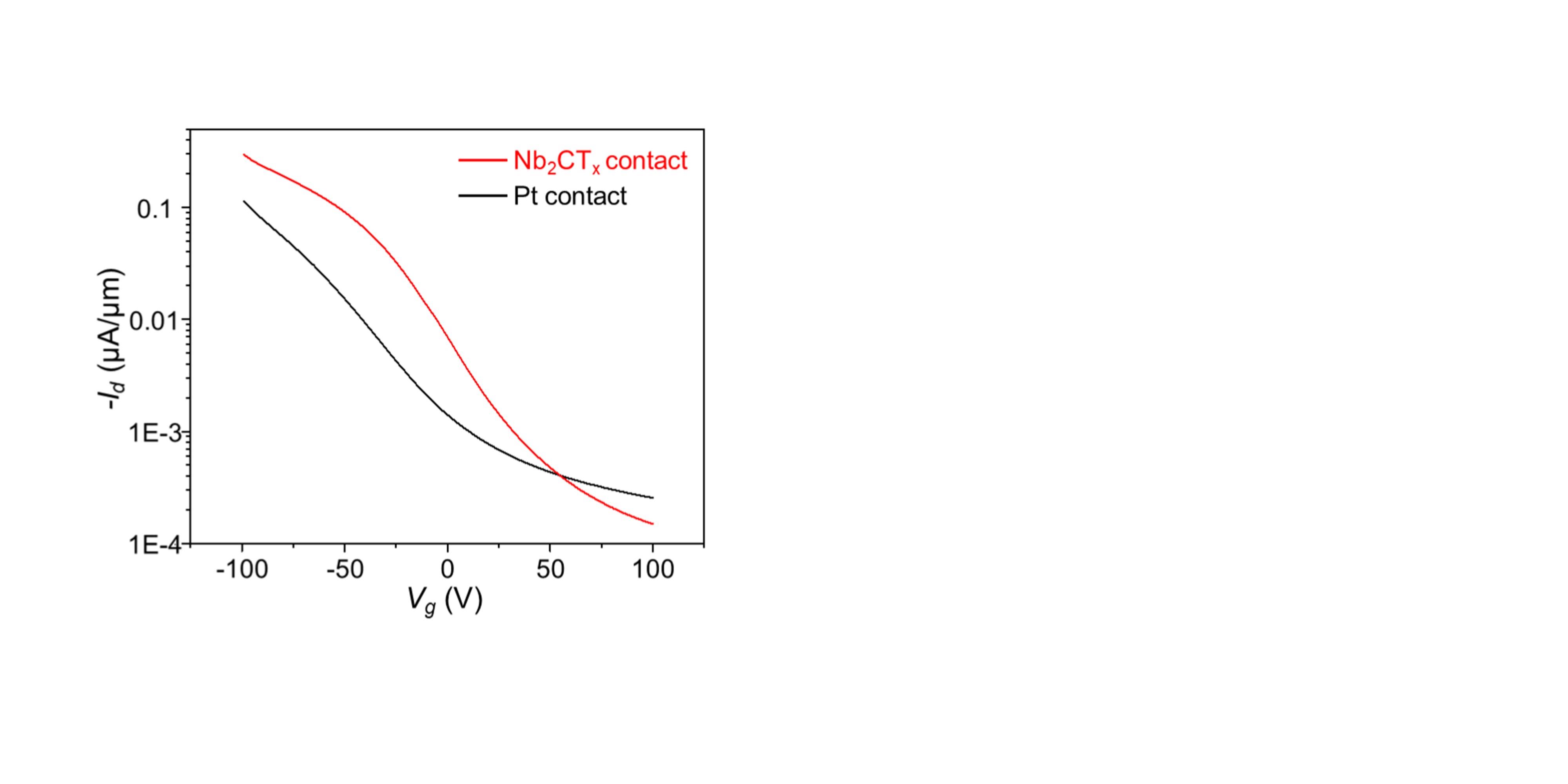


Figure S14. Comparison of transfer characteristics for 2H-MoTe_2_ transistors with Nb_2_CT_x_ and Pt contacts.


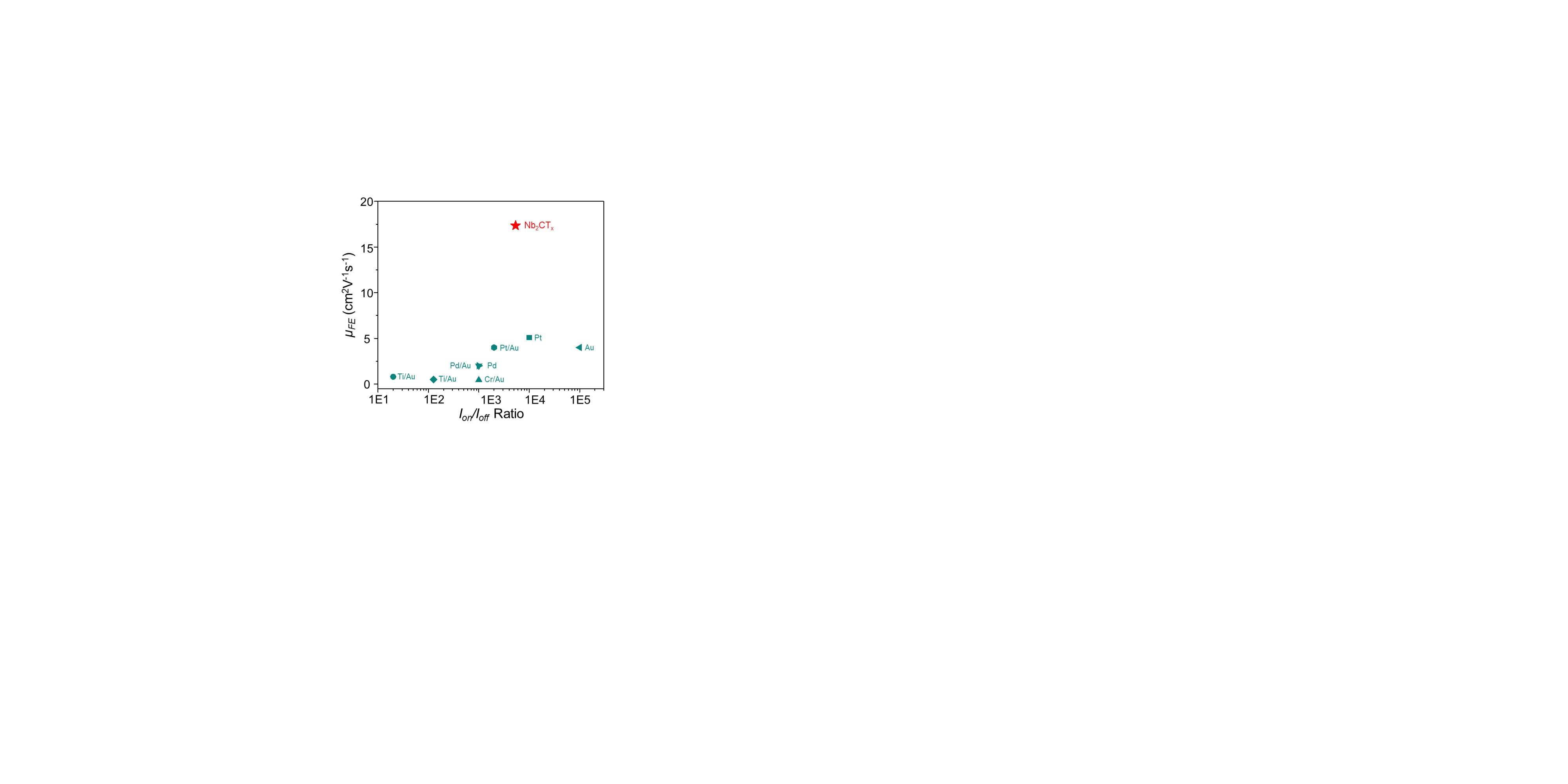


Figure S15. Comparison of the *I_on_/I_off_* ratio and the *μ_FE_* values with the reported metal/*p*-type MoTe_2_ (via CVD) transistors. ^[3-10]^


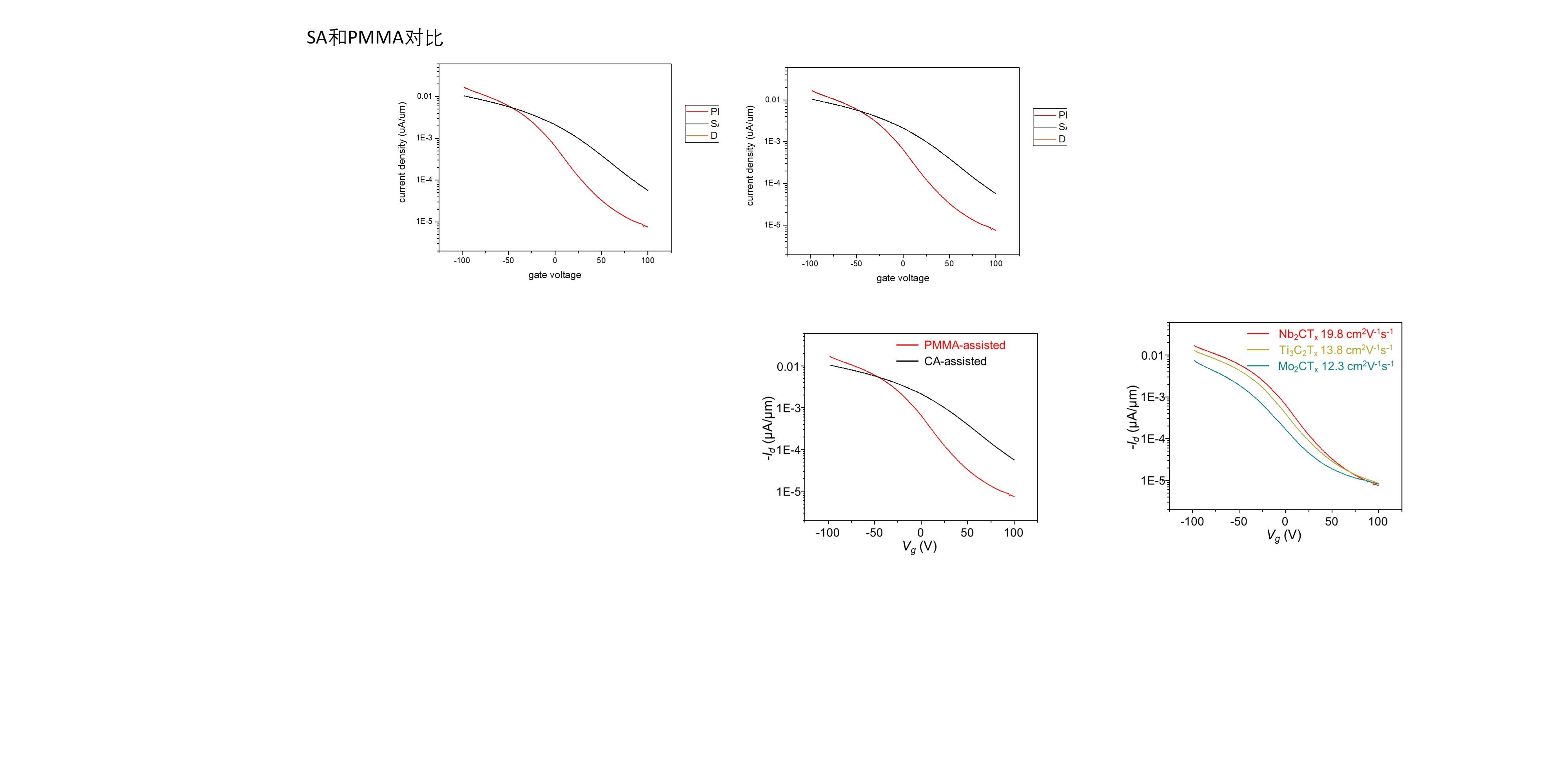


Figure S16. Comparison of transfer characteristics for 2H-MoTe_2_ transistors fabricated via PMMA-assisted and CA-assisted transfer methods.

Table S1. Device performance comparison of CVD‑2H-MoTe_2_ with Nb_2_CT_x_ and traditional metal contacts

| Contact materials | *I_on_/I_off_* ratio | *μ_FE_* (cm^2^ V^-1^ s^-1^) | References |
| --- | --- | --- | --- |
| Pt | 10000 | 5.1 | ^[3]^ |
| Ti/Au | 20 | 0.8 | ^[4]^ |
| Cr/Au | 1000 | 0.44 | ^[5]^ |
| Pd | 1000 | 2 | ^[6]^ |
| Ti/Au | 126 | 0.5 | ^[7]^ |
| Au | 100000 | 4 | ^[8]^ |
| Pd/Au | 1000 | 2 | ^[9]^ |
| Pt/Au | 2000 | 4 | ^[10]^ |
| Nb_2_CT_x_ | ~5300 | ~17 | This work |

**References**

[1] Tianchao Guo, Xiangming Xu, Chen Liu, Yizhou Wang, Yongjiu Lei, Bin Fang, Lin Shi, Hang Liu, Mrinal K. Hota, Hala A. Al-Jawhari, Xixiang Zhang, H. N. Alshareef, ACS Nano 2023, 17, 8324; Zhixiong Liu, Jehad K. El-Demellawi, Osman M. Bakr, Boon S. Ooi, H. N. Alshareef, ACS Nano 2022, 16, 7904; Dhinesh Babu Velusamy, Jehad K. El-Demellawi, Ahmed M. El-Zohry, Andrea Giugni, Sergei Lopatin, Mohamed N. Hedhili, Ahmed E. Mansour, Enzo Di Fabrizio, Omar F. Mohammed, H. N. Alshareef, Adv. Mater. 2019, 31, 1807658.

[2] Xiaolong Xu, Shulin Chen, Shuai Liu, Xing Cheng, Wanjin Xu, Pan Li, Yi Wan, Shiqi Yang, Wenting Gong, Kai Yuan, Peng Gao, Yu Ye, L. Dai, J. Am. Chem. Soc. 2019, 141, 2128.

[3] Seunguk Song, Aram Yoon, Sora Jang, Jason Lynch, Jihoon Yang, Juwon Han, Myeonggi Choe, Young Ho Jin, Cindy Yueli Chen, Yeryun Cheon, Jinsung Kwak, Changwook Jeong, Hyeonsik Cheong, Deep Jariwala, Zonghoon Lee, S.-Y. Kwon, Nat. Commun. 2023, 14, 4747.

[4] Rui Ma, Huairuo Zhang, Youngdong Yoo, Zachary Patrick Degregorio, Lun Jin, Prafful Golani, Javad Ghasemi Azadani, Tony Low, James E. Johns, Leonid A. Bendersky, Albert V. Davydov, S. J. Koester, ACS Nano 2019, 13, 8035.

[5] Yong Ju Park, Ajit K. Katiyar, Anh Tuan Hoang, J.-H. Ahn, Small 2019, 15, 1901772.

[6] Qi Zhang, Xue-Feng Wang, Shu-Hong Shen, Qi Lu, Xiaozhi Liu, Haoyi Li, Jingying Zheng, Chu-Ping Yu, Xiaoyan Zhong, Lin Gu, Tian-Ling Ren, L. Jiao, Nat. Electron. 2019, 2, 164.

[7] Xiang Zhang, Zehua Jin, Luqing Wang, Jordan A. Hachtel, Eduardo Villarreal, Zixing Wang, Teresa Ha, Yusuke Nakanishi, Chandra Sekhar Tiwary, Jiawei Lai, Liangliang Dong, Jihui Yang, Robert Vajtai, Emilie Ringe, Juan Carlos Idrobo, Boris I. Yakobson, Jun Lou, Vincent Gambin, Rachel Koltun, P. M. Ajayan, ACS Appl. Mater. Interfaces 2019, 11, 12777.

[8] Ji Ho Sung, Hoseok Heo, Saerom Si, Yong Hyeon Kim, Hyeong Rae Noh, Kyung Song, Juho Kim, Chang-Soo Lee, Seung-Young Seo, Dong-Hwi Kim, Hyoung Kug Kim, Han Woong Yeom, Tae-Hwan Kim, Si-Young Choi, Jun Sung Kim, M.-H. Jo, Nat. Nanotechnol. 2017, 12, 1064.

[9] Xiaolong Xu, Shuai Liu, Bo Han, Yimo Han, Kai Yuan, Wanjin Xu, Xiaohan Yao, Pan Li, Shiqi Yang, Wenting Gong, David A. Muller, P. Gao, Yu Ye, L. Dai, Nano Lett. 2019, 19, 6845.

[10] Xiaowen Zhang, Maolin Chen, Ning Zhao, Chen Liu, Yuxuan Huang, Junzhu Li, Meng Tang, Aitian Chen, Hang Liu, Xiangpeng Ou, Dongxing Zheng, Udo Schwingenschlögl, Yating Wan, Bo Tian, X. Zhang, Appl. Phys. Lett. 2025, 126, 163102.
